# Peripheral blood mononuclear cell mitochondrial dysfunction in acute alcohol‐associated hepatitis

**DOI:** 10.1002/ctm2.1276

**Published:** 2023-05-25

**Authors:** Annette Bellar, Nicole Welch, Jaividhya Dasarathy, Amy Attaway, Ryan Musich, Avinash Kumar, Jinendiran Sekar, Saurabh Mishra, Yana Sandlers, David Streem, Laura E Nagy, Srinivasan Dasarathy

**Affiliations:** ^1^ Department of Inflammation and Immunity Lerner Research Institue, Cleveland Clinic Cleveland Ohio; ^2^ Department of Gastroenterology and Hepatology Cleveland Clinic Cleveland Ohio; ^3^ Departnent of Pulmonary Medicine Cleveland Clinic Cleveland Ohio; ^4^ Department of Chemistry Cleveland State University Cleveland Ohio; ^5^ Department of Psychiatry and Psychology Cleveland Clinc Lutheran Hospital Cleveland Ohio

**Keywords:** alcohol‐associated hepatitis, immune senescence, intermediary metabolites, mitochondrial oxidation, peripheral blood mononuclear cells, single‐cell RNA sequencing, telomere

## Abstract

**Background:**

Patients with acute alcohol‐associated hepatitis (AH) have immune dysfunction. Mitochondrial function is critical for immune cell responses and regulates senescence. Clinical translational studies using complementary bioinformatics‐experimental validation of mitochondrial responses were performed in peripheral blood mononuclear cells (PBMC) from patients with AH, healthy controls (HC), and heavy drinkers without evidence of liver disease (HD).

**Methods:**

Feature extraction for differentially expressed genes (DEG) in mitochondrial components and telomere regulatory pathways from single‐cell RNAseq (scRNAseq) and integrated ‘pseudobulk’ transcriptomics from PBMC from AH and HC (*n* = 4 each) were performed. After optimising isolation and processing protocols for functional studies in PBMC, mitochondrial oxidative responses to substrates, uncoupler, and inhibitors were quantified in independent discovery (AH *n* = 12; HD *n* = 6; HC *n* = 12) and validation cohorts (AH *n* = 10; HC *n* = 7). Intermediary metabolites (gas‐chromatography/mass‐spectrometry) and telomere length (real‐time PCR) were quantified in subsets of subjects (PBMC/plasma AH *n* = 69/59; HD *n* = 8/8; HC *n* = 14/27 for metabolites; HC *n* = 13; HD *n* = 8; AH *n* = 72 for telomere length).

**Results:**

Mitochondrial, intermediary metabolite, and senescence‐regulatory genes were differentially expressed in PBMC from AH and HC in a cell type–specific manner at baseline and with lipopolysaccharide (LPS). Fresh PBMC isolated using the cell preparation tube generated optimum mitochondrial responses. Intact cell and maximal respiration were lower (*p* ≤ .05) in AH than HC/HD in the discovery and validation cohorts. In permeabilised PBMC, maximum respiration, complex I and II function were lower in AH than HC. Most tricarboxylic acid (TCA) cycle intermediates in plasma were higher while those in PBMC were lower in patients with AH than those from HC. Lower telomere length, a measure of cellular senescence, was associated with higher mortality in AH.

**Conclusion:**

Patients with AH have lower mitochondrial oxidative function, higher plasma TCA cycle intermediates, with telomere shortening in nonsurvivors.

## Graphical abstract

- Alcohol associated hepatitis is a severe form of liver disease with immune dysfunction that contributes to risk of infections and mortality.
- Immune cells, including peripheral blood mononuclear cells, responses include cell proliferation, cytokine and immunoglobulin secretion, all of which are high energy requiring functions that depend on mitochondrial oxidative function.
- Since mitochondrial responses are sensitive to cell processing protocols, we developed an optimal approach to isolate peripheral blood mononuclear cells for functional assays.
- Complementary multiomics analyses of single cell RNA sequencing data and experimental validation studies showed mitochondrial oxidative dysfunction, intermediary metabolite alterations and telomere shortening in peripheral blood mononuclear cells in patients with alcohol‐associated hepatitis.

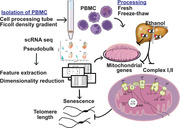

## 1 INTRODUCTION

Acute alcohol‐associated hepatitis (AH) is one of the most severe forms of liver diseases with poor clinical outcomes. Immune dysfunction in AH contributes to both liver injury and risk of infection with consequent adverse clinical outcomes.^1^ Peripheral blood mononuclear cells (PBMC) are a heterogeneous group of circulating cells that contribute to host defence against pathogens and secrete pro‐ and anti‐inflammatory cytokines.^2^ Responses to lipopolysaccharide (LPS) have been evaluated as noninvasive biomarkers of PBMC function in a number of diseases including AH.^3^, ^4^ Functional responses require mRNA translation, to synthesise a number of signalling and secretory proteins, a high‐energy requiring cellular function that depends on mitochondrial oxidative function.^5^, ^6^, ^7^ Bioenergetic dysfunction with reduced ATP content has been reported in multiple organs in response to ethanol exposure and in AH.^8^, ^9^ Consistently, defects in mitochondrial oxidation, a major source of ATP synthesis in most cells, have been reported in liver and skeletal muscle^8^, ^9^, ^10^ in response to ethanol exposure; however, functional responses of electron transport chain (ETC) components of PBMC in AH are currently not known. Altered mitochondrial intermediary metabolites contribute to oxidative dysfunction,^9^ but whether intermediary metabolites in peripheral immune cells are altered during AH is also not known. Mitochondrial dysfunction and consequent oxidative stress contribute to both replicative and nonreplicative senescence with telomere length attrition.^11^, ^12^ Increased free radical generation due to mitochondrial oxidative dysfunction causes cell senescence in a number of acute and chronic diseases,^13^ but whether PBMC from patients with AH have evidence of senescence is not known.

Unlike unbiased data, metabolite quantification, and some molecular validation studies that can be done in frozen cells, functional responses, including mitochondrial oxidation, require live cells or reviving frozen cells, and are influenced by the processing method for tissue/cell isolation.^14^, ^15^ Recent data suggest that processing of frozen cells can provide adequate functional responses, but direct comparisons of fresh and frozen cells or the impact of processing protocols^15^, ^16^ have not been reported in PBMC in acute illness. Our studies in PBMC from patients with AH and heavy drinkers without liver disease (HD) are, therefore, of high clinical and translational significance. Even though defective PBMC responses in AH have been reported,^17^, ^18^, ^19^ most studies in human subjects report the use of frozen PBMC^19^, ^20^, ^21^ and mitochondrial oxidative responses (including ETC component function) in PBMC from these patients have not been reported. Recent data on whole PBMC transcriptomics from patients with AH showed a significant increase in expression of components of the ETC that was interpreted as an increased ATP synthesis.^20^ However, changes in transcriptomics do not necessarily translate into functional consequences and may be an adaptive response to underlying mitochondrial dysfunction since ethanol and its metabolites are known inhibitors of mRNA translation.^9^, ^22^ We evaluated published single‐cell RNAseq (scRNAseq) data of PBMC^21^ followed by integration of these data across cells to generate a ‘pseudobulk’ dataset that allowed us to simultaneously assess individual cellular transcriptomic responses to determine global PBMC mitochondrial oxidative function, intermediary metabolite concentrations, and senescence.

There are limited data on experimental validation of transcriptomic responses. In the present prospective studies, we determined the optimum processing method and the impact of freezing on mitochondrial oxidative function of PBMC from patients with AH, HD and healthy controls (HC). We then identified the specific defects in the mitochondrial ETC complexes and related them to our bioinformatics studies from scRNA sequencing. Our approach of simultaneously using scRNA and integrated pseudobulk analyses allows us to integrate RNAseq data with functional outcomes while simultaneously laying the foundation for future studies to analyse contributions of individual cell types to unique and integrated functional responses. Complementary intermediary metabolite concentrations in plasma and PBMC and telomere length were then related to clinical outcomes in AH. Our data provide a comprehensive analysis of mitochondrial responses in PBMC and provide potential mechanistic insights into peripheral immune senescence in AH including differential contributions of cell types within the PBMC population.

## 2 PATIENTS AND METHODS

All human studies were performed after obtaining a written informed consent, approved by the Institutional Review Board (IRB) at the Cleveland Clinic (IRB 19–1041) and in accordance with the ethical principles for medical research as outlined in the Declaration of Helsinki. The study is registered on clinicaltrials.gov (NCT04088370).

In brief, subjects were recruited at the Cleveland Clinic from the inpatient and outpatient services. Healthy subjects and heavy drinking controls were enrolled by advertisement and from the clinical research unit at the Cleveland Clinic. Subjects with AH: a clinical diagnosis of AH was made by the clinicians and evaluated by the clinical co‐authors (JD, NW, SD) based on medical history, physical examination, and laboratory results including biochemical values and/or liver biopsy as well as recent drinking history (> 40 g/day on average for women and > 60 g/day on average for men within 8 weeks) as used in the Defeat Alcoholic SteatoHepatitis study.^23^ Healthy control: No known chronic diseases or treatment for chronic diseases; INR < 1.4 and total bilirubin levels must be < 3. No prior history of known alcoholic liver disease and alcohol consumption < 7 drinks per week for women and 14 drinks for men. Heavy drinking controls: subjects who satisfied the alcohol consumption criteria for AH but with no evidence of liver disease on clinical, biochemical and imaging criteria. After screening for the inclusion and exclusion criteria, patient‐reported outcomes using the physical function and fatigue domain of the NIH‐validated Patient‐Reported Outcomes Measurement Information Systems (PROMIS®) questionnaires^24^ were documented. Whole blood was obtained and processed for isolation and evaluation of PBMC function, metabolic and telomere length studies.

Analyses of scRNAseq in PBMC from patients with AH and HC were complemented by experimental studies to determine the impact of processing of PBMC on mitochondrial oxidative function, mitochondrial responses in AH, HD and HC; tricarboxylic acid (TCA) cycle intermediary metabolite concentrations; and telomere length in the different groups of subjects.

### 2.1 Blood collection and isolation of PBMC

Venous blood from the upper arm was collected from patients in EDTA‐coated vials and were centrifuged initially at 800 × *g* at room temperature for 15 min to separate the plasma and white blood cell layer. Plasma was collected from EDTA coated vials and frozen at −80°C for analyses. The white blood cells were collected and PBMC separated either by Ficoll gradient or cell processing tube (CPT) methods. Blood was gently layered on a Ficoll gradient or collected in CPT tubes and centrifuged for 30 min at 1800 × *g* with no brake. The collected PBMC were washed twice with phosphate buffered saline at room temperature and resuspended in Roswell Park Memorial Institute (RPMI) medium. Viable cells were counted using trypan blue exclusion of which 1.5 million cells used for mitochondrial studies and the remaining cells were frozen in freezing media (90% RPMI to 10% DMSO) for studies on frozen cells. The effect of processing of PBMC (separation protocol) or freeze‐thaw on mitochondrial oxidative function was evaluated in PBMC from the same subjects.

### 2.2 Single‐cell RNA sequencing and analyses

Clinical and procedural details for scRNAseq have been previously reported.^21^ For these studies, feature extraction and analyses including single‐cell expression as well as integrated pseudobulk analyses were performed for analyses in the total PBMC pool from each subject. Cryopreserved PBMC were used for scRNAseq from healthy controls and patients with acute alcohol related hepatitis (*n* = 4 each). For cryopreservation, PBMC were separated by density gradient centrifugation, resuspended in freezing media (50% culture media, 40% FBS, 10% DMSO) and allowed to freeze slowly to −80°C. For LPS responses, cells were thawed at 37°C, added to warm culture media, centrifuged, resuspended and cultured in 96‐well plates in a humidified atmosphere (5% CO2, 37°C). After 18 h, cells were washed with media and challenged with 100 pg/mL LPS for 24 h. Cells were washed, resuspended and counted for 10x sequencing (Chromium v3.1). Libraries were generated per manufacturer instructions and quantified using an Agilent Bioanalyzer (Agilent Technologies, Santa Clara, CA), pooled and sequenced as reported earlier.^21^

### 2.3 Integrating single‐cell RNA sequencing to generate dot plots for pseudobulk analyses

Integration of scRNAseq data was performed using the single‐cell Seurat object that is included in the Seurat package for visualisation. Using this approach, the colour of the dots represents the average expression of a gene for each cell type group and the size of the dot represents the percentage of that cell type group that expresses that gene (count > 0). The colouration scale is Z‐score normalised for each gene so that the average is set to 0 and the values of 1, 2, 3 are ‘X standard deviations away from the “average” expression level for Gene A’.

### 2.4 Bioinformatics analyses

Differentially expressed mitochondrial genes in clusters of mononuclear cells based on expression of antigen markers were quantified as previously reported.^25^ Changes in mitochondrial and telomere regulatory genes in each cluster were identified by matching with the Mitocarta3.0 and TelNet databases^26^, ^27^ followed by curation and differential expression of each subcomponent within the ETC. Since datasets for ETC components and oxidative phosphorylation have shared and unique genes, we used both datasets to provide a comprehensive analysis of mitochondrial oxidative function including components of the ETC (MitoCarta 3.0),^26^ and telomere‐regulation (TelNet)^27^ databases were matched against the expressions and enrichment of genes in each cluster of the PBMC in the basal state and in response to LPS.

Sequencing data were aligned to the Human genome (GRC38, release 93) using Cellranger (v3.0.2) and gene expression and clustering analyses were performed using Seurat (3.1.1).^28^

For experimental validation of the unbiased data, mitochondrial oxidative function was quantified using high‐sensitivity respirometry, and telomere length determined by real‐time PCR.

### 2.5 Optimisation of PBMC processing for mitochondrial oxidative function

Effects of processing for PBMC isolation using CPT or Ficoll density gradient from the same subjects on mitochondrial oxidative function were quantified. Since PBMC isolation protocols are time consuming, frozen cells have been used for functional responses by others.^29^, ^30^ Since cellular functional responses are ATP dependent, we evaluated mitochondrial oxidative function in freshly isolated and previously frozen PBMC collected at the same time from the same subject (7 healthy controls and 9 AH). In this protocol, part of the PBMC were frozen, revived the next day, and mitochondrial oxidative function was quantified in intact PBMC.

Mitochondrial respiration in both intact and permeabilised cells was measured at 37°C on a high‐sensitivity fluororespirometer (Oroboros, Innsbruck, Austria) using substrate, inhibitor titration (SUIT) protocols previously reported by us^9^, ^25^ and described in brief below .

#### 2.5.1 Intact cell respiration

Respiration rates (oxygen flux) were expressed per million live cells. Intact cell respiration of PBMCs was measured in cells suspended in RPMI media. ATP‐linked respiration, proton leak, and maximum respiratory capacity measured in response to the protonophore, carbonyl cyanide‐4‐(tri fluoromethoxy) phenylhydrazone (FCCP), and the response to complex I inhibitor, rotenone, and Antimycin A, an inhibitor of cytochrome c reductase, were quantified. In addition, intact cell respiration was measured in PBMCs that were frozen and those results were compared to the results of the same patients fresh cells. Cells were frozen at −80°C for at least 24 h then thawed and resuspended in RPMI media. Live cells were counted again to ensure proper cell number.

#### 2.5.2 Permeabilised cell respiration

Standard SUIT protocols were used to quantify mitochondrial function in digitonin permeabilised PBMCs suspended in mitochondrial respiration medium, MiR05 (0.5 mM EGTA, 3 mM MgCl_2_.6H2O, 60 mM potassium lactobionate, 20 mM taurine, 10 mM KH_2_PO_4_, 20 mM HEPES, 110 mM sucrose, 1 g/L bovine serum albumin, pH 7.1). The sequence, dose and details of these experiments have been described by us previously.^9^ In brief, malate, pyruvate, and glutamate were provided as complex I substrates, followed by succinate as a complex II substrate, to quantify oxidative phosphorylation. ATP synthesis and oxidative phosphorylation were subsequently quantified in response to ADP administration. Oxidation and phosphorylation were then uncoupled using a protonophore, FCCP, to measure the maximum oxidation capacity. Rotenone‐sensitive respiration (uncoupled complex I rate of oxygen consumption) and rotenone‐insensitive respiration (uncoupled complex II rate of oxygen consumption) were measured. Nonmitochondrial respiration was measured using Antimycin A‐insensitive oxygen consumption. Uncoupled complex IV oxidation was calculated as the difference between the azide‐insensitive and the tetramethyl phenylene diamine (TMPD) with ascorbate oxygen consumption rates.

### 2.6 Intermediary metabolite quantification

Intermediary metabolites in plasma and PBMC were quantified as previously described.^25^, ^31^ Plasma for these analyses were collected from EDTA coated tubes due to dilution with Ficoll and CPT methods. In brief, GC‐MS analysis was performed with Agilent 5977 mass spectrometer coupled to a 7890 B gas chromatograph fitted with a 7693 autosampler and a DB‐5 ms column (Agilent, Santa Clara, CA, USA). The GC‐MS was operated as electron impact (EI)/single ion monitoring (SIM) mode. Labelled internal standards for each of the measured metabolites were used for quantification. The temperature program was as follows: 80°C hold for 2 min followed by an increase of 15°C/min to a total heat of 305°C held for 3 min. Calibrations curves with at least six points were obtained by plotting the metabolite/internal standard peak ratio versus the metabolite concentrations in spiked plasma and PBMC followed by linear regression analysis. The criteria for acceptance were set as a correlation coefficient *r* ^2^ > 0.99. Carryover was examined by extracting spiked samples with a high level of analytes followed by GC‐MS runs of these samples and blanks.

### 2.7 Telomere length measurment and calculations

Genomic DNA was isolated from healthy controls and patients with alcoholic hepatitis using the Qiagen DNeasy® Blood and Tissue Kit (Qiagen Inc. Germantown, MD) using the manufacturer protocol. In brief, frozen PBMCs were thawed and centrifuged to remove the freezing medium and resuspended in 200 μL of sterile phosphate buffered saline and 200 μL of Buffer AL. Samples were vortexed and then incubated at 56°C for 10 min. Absolute ethanol (200μL) was added and the solution was vortexed, placed into a DNeasy® Mini spin column and centrifuged at 6000 × *g* for 1 min. Buffers were added to the columns per manufacturer guidelines DNA was eluted in 100 μL of Buffer AE. Genomic DNA was quantified and purity was measured using a NanoDrop™ Spectrophotometer (ThermoFisher Scientific, Waltham, MA).

Telomere length was measured using the SYBR Green Master Mix (ThermoFisher Scientific, Waltham, MA). Each patient DNA sample was amplified with a human telomere primer and the human acidic ribo phos primer as the single copy reference (SCR) gene as previously reported by others. The DNA sample, primer, SYBR® Green Master Mix and nuclease free water were mixed and each PCR was performed in triplicate along with positive and negative controls. The qPCR program was run as follows Initial Denaturation step 95°C for 10 min followed by 40 cycles of 95°C for 20 s (denaturation), 52°C for 20 s (annealing), and 72°C for 45 s (extension) followed by a melting curve.

The average of each triplicate run was calculated to obtain the average telomere and SCR CT values. The average telomere and SCR CT of each patient sample was subtracted from the average telomere CT or SCR of the reference sample to calculate the ΔCq(Tel) and ΔCq(SCR). ΔCq(SCR) values were subtracted from ΔCq(Tel) for each patient to generate the ΔΔCq(Tel). Relative telomere length was calculated using the following equation 2^−ΔΔCq(Tel)^ . Finally total telomere length was calculated by multiplying the 2^−ΔΔCq(Tel)^ of each patient by the reference sample telomere length.

For our analysis, we log‐transformed telomere length followed by standardising the results by Z‐score as previously described.^32^, ^33^ This method minimises the impact of potential batch effects across multiple samples.^32^, ^33^ We then used Youden's optimal cut‐point criteria to calculate a Z‐score cutoff based on the sensitivity and specificity of telomere length with mortality as the dependent variable. Multivariate Cox proportional hazard analysis was then performed for the Z‐score cutoff (adjusted for age, sex and body mass index‐BMI). These methods have been utilised to assess the strength of biomarkers,^34^ and telomeres are increasingly being considered as a biomarker of ‘biological age’.^35^

### 2.8 Statistical analyses

Descriptive data were expressed as ratios, proportions and mean ± standard deviation (SD). Qualitative variables were compared using the chi square test. Quantitative variables were compared using an analysis of variance with Bonferroni post‐hoc analysis or Student's t test for independent variables. Significance was set at *p* < .05.

The numbers of subjects including those shared across various studies is shown in Figure S1 and Tables S1 and S2.

## 3 RESULTS

The clinical, demographic, bioinformatics, and laboratory data of the complete cohort of HC, HD and AH subjects and subgroups of subjects in each study showed that, in addition to liver related perturbations, higher fatigue‐related symptoms, and mortality were observed only in patients with AH (Tables 1 and S1–S27). Shared and unique analyses/studies in different cohorts are shown as UpSet plots (Figure S1). High MELD score and its components, and higher BMI were associated with mortality in AH (Table S23).

**TABLE 1:** Clinical and demographic findings of subjects.

	HC	HD	AH
Number	31	11	81
Male:female	16:15	6:5	52:29
Age in years (mean ± SD)	41.94 ± 13.99	43.64 ± 14.04	47.93 ± 11.55
Alive:dead	31:0	11:0	40:41^a^
Race W:AA:A:U	21:1:6:3	9:1:0:1	64:12:1:4
Body mass index (kg/m^2^)	24.64 ± 4.57	28.20 ± 5.31	28.62 ± 6.16^b^
Total leucocyte count (x1000/μL)	6.68 ± 3.07	6.16 ± 2.42	13.70 ± 7.63^c^, ^d^
Platelet (x1000/μL)	260.96 ± 69.19	239.90 ± 67.43	125.44 ± 71.69^a^
Alanine amino transferase (U/L)	18.78 ± 8.49	29.40 ± 17.53	53.60 ± 44.93^c^
Aspartate aminotransferase (U/L)	20.93 ± 7.99	27.60 ± 14.44	130.07 ± 97.58^a^
Serum albumin (g/dL)	4.46 ± 0.32	4.43 ± 0.32	2.97 ± 0.54^a^
Serum total protein (g/dL)	7.21 ± 0.40	7.24 ± 0.41	5.68 ± 0.88^a^
Bilirubin (mg/dL)	0.59 ± 0.62	0.35 ± 0.12	15.66 ± 12.01^a^
Alkaline phosphatase (U/L)	64.59 ± 22.74	77.90 ± 38.79	173.91 ± 128.27^c^, ^e^
Serum creatinine (mg/dL)	0.85 ± 0.16	0.80 ± 0.11	1.85 ± 1.65^f^
Blood urea nitrogen (mg/dL)	13.48 ± 3.74	11.50 ± 3.31	29.62 ± 26.01^b^, ^e^
Serum sodium (mmol/L)	139.26 ± 2.44	138.30 ± 2.67	134.11 ± 5.01^c^, ^e^
International normalised ratio	1.01 ± 0.09^e^	1.06 ± 0.20^f^	1.87 ± 0.60^a^
Steroids (Y:N)	1:30	0:11	47:34^a^
Tobacco use Yes:quit:never:unknown	0:1:12:18	1:1:2:7	19:31:25:6
Antibiotics (Y:N)	8:23	3:8	76:5
Liver‐specific outcomes			
LOS in days (mean ± SD)			18.1 ± 17.5 (0–92)
GI bleed (Y:N)			33:48
Ascites (Y:N)			56:25
Average number of hospitalisations (mean ± SD) (range)			2.0 ± 2.6 (0–10)
Hepatic encephalopathy (Y:N)			59:22
Cirrhosis (Y:N)			56:25
Mean survival from enrolment in days (mean ± SD) (range)			433.0 ± 378.3 (1–1755)
MELD score (mean ± SD) (range)			26.28 ± 8.91 (8.33–52.82)
UTI (Y:N)			1:80
Sepsis (Y:N)			8:73
Pneumonia (Y:N)			76:5
SBP (Y:N)			16:65

Abbreviations: A: Asian, AA: African American, AFP: alpha fetoprotein, AH: alcohol‐associated hepatitis, GI: gastrointestinal; HC: healthy control, HD: healthy heavy drinker, LOS: Length of Stay, MELD: model for end stage liver disease, N: no, SBP: spontaneous bacterial peritonitis, SD: standard deviation, U: unknown, UTI: urinary tract infection, W: White, Y: Yes.

^a^ *p* < .001 AH vs. HC, HD.

^b^ *p* < .01 AH vs. HC, HD.

^c^ *p* < .001 AH vs. HC.

^d^ AH vs. HD *p* < .01.

^e^ AH vs. HD *p* < .05.

^f^ AH vs. HC *p* < .01.

^g^ AH vs. HC *p* < .05.

### 3.1 Transcriptomic responses

**FIGURE 1:**
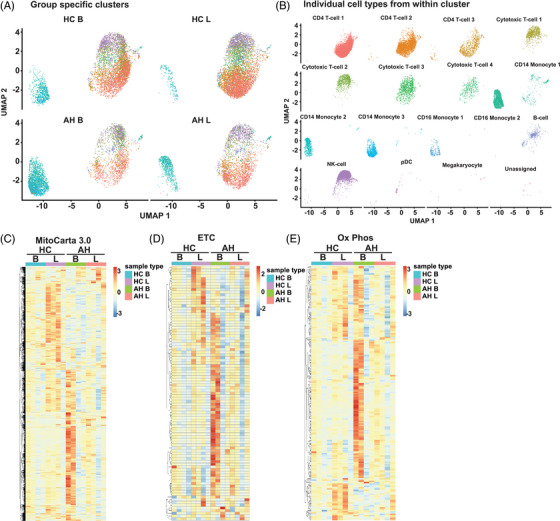
Single‐cell RNA sequencing clustering of mitochondrial genes. Feature extraction of mitochondrial genes was followed by Uniform Manifold Approximation and Projection (UMAP) and Seurat analysis to identify clustering by specific cell type based on mitochondrial gene expression in peripheral blood mononuclear cells from four groups of patients: healthy control (HC) or alcohol‐associated hepatitis (AH) subjects at baseline (B) and in response to stimulation by lipopolysaccharide (LPS). (A) Cell clusters (all types or subtypes by known markers) irrespective of subject group (AH/HC unstimulated/stimulated with LPS combined). (B) Cell clusters by mitochondrial gene expression in the 4 patient groups. (C–E) Supervised heatmaps of average expression on pseudobulk transcriptomics generated by of single‐cell RNA sequencing (scRNAseq). (C) Mitochondrial targeted genes. (D) Electron transport chain (ETC) genes. (E) Oxidative phosphorylation (OXPHOS) consolidation genes. Data from *n* = 4 in each group.

Clinical, laboratory findings, and scRNAseq expression in the four groups of subjects (HC and AH in the unstimulated/basal state and in response to stimulation with LPS) have been published previously.^21^ Feature extraction of mitochondrial genes on uniform manifold approximation and projection (UMAP) plots showed that PBMC cell types clustered by disease and LPS responses of mitochondrial gene expression with the greatest nonoverlap of monocytes and nonmonocytes (Figure 1A). Within each patient population (HC untreated vs. LPS, AH untreated vs. LPS) also, distinct clustering was noted showing differential mitochondrial gene expression responses in a cell‐specific and group‐specific manner (Figure 1B). Supervised heatmaps of the pseudobulk dataset showed distinct patterns of differentially expressed genes (DEG) among all mitochondrial genes across the four groups of subjects (Figure 1C; DEGs in Table S3). Since our focus was on mitochondrial oxidative function, we then generated UMAP plots from scRNAseq (Figure S2) and supervised heatmaps of components of the ETC/oxidative phosphorylation DEGs from the pseudobulk dataset (Figure 1D and E, DEGs in Tables S4 and S5). UMAP plots of components of the ETC (complexes I–V) involved in oxidative phosphorylation in the four groups of subjects showed that monocytes and nonmonocytes were nonoverlapping with the most changes in expression of complexes I and III (Figure S2). Unsupervised heatmaps of the pseudobulk datasets of mitochondrial and ETC/oxidative phosphorylation DEGs (Figure S3A and B; Tables S6 and S7) were similar to supervised heatmaps (Figure 1C–E) in terms of separation of HC and AH, but a similar robust separation on unsupervised analysis was not observed for LPS responses. We then generated a heatmap of average scRNAseq expression of genes in the oxidative phosphorylation pathway in PBMC of HC and AH in the basal, unstimulated state. The average expression patterns on scRNAseq showed clear differences between cell‐type responses, with the most change in expression in monocytes, plasmacytoid dendritic cells (pDC), and megakaryocytes (Figure S3C). These data show that components of the ETC were altered in AH.

**FIGURE 2:**
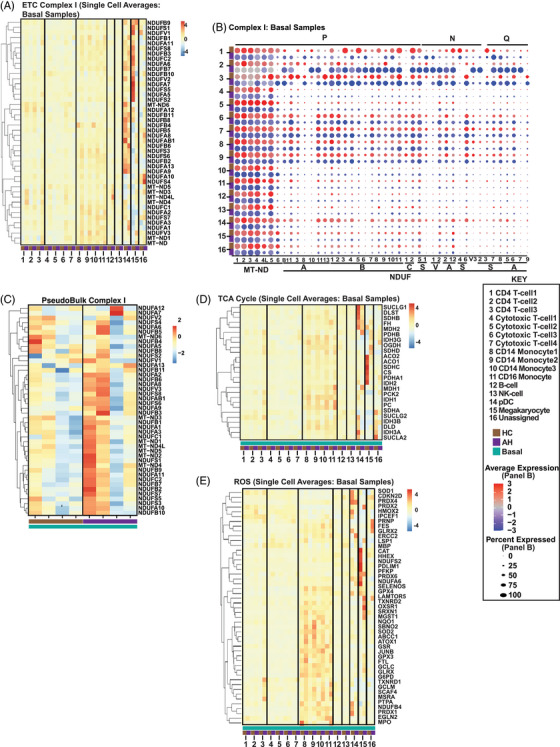
Integrated analyses of mitochondrial function genes. Single‐cell RNA sequencing (scRNAseq) analyses of comparative average transcript expression of genes in peripheral blood mononuclear cells from patients with alcohol‐associated hepatitis (AH) or healthy control (HC) subjects at baseline. (A) Heatmap of average expression of single‐cell analysis of all electron transport chain (ETC) complex I components. (B) Schematic dot‐plot representation of scRNAseq data of comparative average expression of all ETC complex I components with Proton Pumping (P), NADH oxidase (N), and ubiquinone reductase (Q) modules noted on the top. (C) Heatmap of average expression of complex I components at baseline on pseudobulk transcriptomics generated by consolidation of scRNAseq. (D, E) Heatmap of average expression of single‐cell analysis of tricarboxylic acid (TCA) cycle and free radical (reactive oxygen species; ROS) genes. Data from *n* = 4 in each group. Full Gene Names for Panel 2B: MT‐ND1, MT‐ND2, MT‐ND3, MT‐ND4, MT‐ND4L, MT‐ND5, MT‐ND6, NDUFAB1, NDUFA1, NDUFA3, NDUFA8, NDUFA10, NDUFA11, NDUFA13, NDUFB1, NDUFB2, NDUFB3, NDUFB4, NDUFB5, NDUFB6, NDUF7, NDUFB8, NDUFB9, NDUFB10, NDUFB11, NDUFC1, NDUFC2, NDUFS5, NDUFS1, NDUFV1, NDUFV2, NDUFA2, NDUFA12, NDUFS4, NDUFS6, NDUFV3, NDUFS2, NDUFS3, NDUFS7, NDUFS8, NDUFA5, NDUFA6, NDUFA7, NDUFA9.

Since complexes within the ETC have a number of components, we then dissected the DEG of the individual ETC components in PBMC from the four groups of subjects (HC/AH, basal/LPS stimulated). To determine cell type DEGs in the PBMC from HC and AH, we generated a heatmap of average scRNAseq expression of components of complex I in the basal, unstimulated state (Figure 2A). The most changes were observed in natural killer cells (NK) and pDC. However, heatmaps for average cell‐type scRNA expression of gene datasets do not account for the percentage of cells expressing the genes of interest. We therefore generated dot plots that allow for simultaneous comparisons of expression and percentage of cells expressing a gene on scRNAseq. We noted cell type–specific and group‐specific (HC or AH in the basal unstimulated state) patterns of differential expression of complex I (Figure 2B). Greatest expression and change in patterns were noted in the proton pumping (P‐module) of complex I in most cell types. However, in the monocytes and pDCs, there were large changes in expression/percentage of cells expressing components of the NADH oxidase (N‐) and ubiquinone reductase (Q‐modules). Similar dot plots were generated for comparison of other groups (Figure S4A–C). Distinct expression patterns for different components of complex I were noted for each comparison, suggesting differences between HC and AH (basal/LPS stimulated). We then tested if there were group‐specific differences across average expressions. We generated a supervised heatmap of a pseudobulk dataset for complex I components in HC and AH in the basal state that was consistent with the previous analyses with different expression patterns in PBMC in these subject groups (Figure 2C). Similar comparative analyses included a supervised heatmap of average scRNA expression in HC and AH in the basal state, 4 dotplots for different group comparisons (basal, LPS stimulated, HC, AH), and a pseudobulk heatmap for complexes II‐V (Figures S5 to S8A–F). An increase in expression of components of complexes I–III was noted in AH compared to HC in the basal state, but responses to LPS identified in HC were not seen with AH. Defects in ETC components can alter metabolic responses and increase free radical (FR) generation. We therefore evaluated genes regulating the TCA cycle, glycolysis, and FR pathways by generating supervised heatmaps of scRNAseq expression of components of these pathways. These pathway‐specific heatmaps again showed most change in expression patterns in the various monocyte subgroups and pDCs (Figures 2D and E and S9A). Supervised heatmaps of pseudobulk datasets for glycolysis, TCA cycle, FR, and antioxidant pathways showed differential expression of components of these pathways in the four subject groups (Figure S9B–E; Tables S8–S11). In summary, scRNAseq and integrated pseudobulk analyses of PBMC responses showed an increase in critical components of complexes in the basal state in AH compared to HC but the increase in expression with LPS was noted in HC but not with AH. Importantly, differentially expressed mitochondrial and ETC component genes were significantly different between monocytes and nonmonocytes.

Since transcriptomics data do not necessarily correspond with functional responses, we experimentally evaluated mitochondrial oxidative function and intermediary metabolite concentrations in PBMC. Since the numbers of cells in each subtypes are small for individual cell type function(s), global responses were measured and pseudobulk analyses were then integrated with functional measurements. Since processing protocols impact cell functions, we first optimised PBMC isolation procedures to determine the impact of the separation and storage protocol on cellular responses. We performed high‐sensitivity respirofluorometry to determine oxygen consumption as readouts for the best PBMC isolation protocol.

### 3.2 Optimum processing of human PBMC for mitochondrial oxidative function studies

Direct comparisons of mitochondrial oxidative function in intact PBMC processed concurrently (*n* = 11 HC; *n* = 4 AH) using CPT versus Ficoll density gradient (Figure S10A and B) showed significantly higher intact cell respiration, ATP‐linked respiration, maximal respiration, and reserve respiratory capacity with CPT compared to Ficoll separation in both AH and HC. Since mitochondrial dysfunction impairs rather than increases oxygen consumption, these data show that for mitochondrial functional studies, CPT is the optimal processing method.

To determine the effect of freezing on mitochondrial oxidative function, fresh PBMC and those frozen for 24 h before revival were studied (Figure S10C and D). Previously frozen cells vs. freshly isolated PBMC showed consistently lower ATP‐linked respiration, maximum respiration, and reserve respiratory capacity.

Having established that fresh PBMC isolated using the CPT protocol was optimum for mitochondrial functional studies, we then evaluated mitochondrial oxidative function in AH and compared it to that in HC/HD.

### 3.3 Mitochondrial oxidative function in AH

**FIGURE 3:**
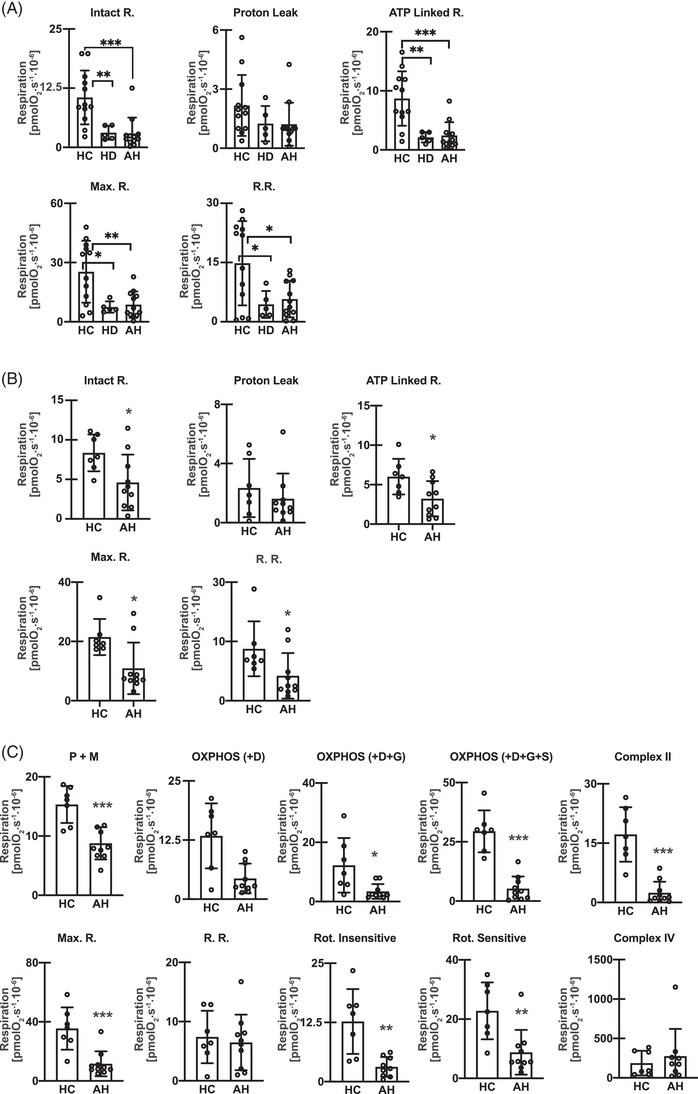
Mitochondrial oxidative dysfunction in patients with alcohol‐associated hepatitis. Oxygen consumption measured using high‐sensitivity respirometry in peripheral blood mononuclear cells (PBMC) from healthy controls (HC), heavy drinking controls (HD), and patients with alcohol‐associated hepatitis (AH) in the same patient in intact and permeabilised cells (in the discovery cohort). (A) Intact cell responses in a discovery cohort including responses to ATP synthetase inhibitor‐oligomycin, uncoupler of oxidative phosphorylation, 2‐[2‐[4‐(trifluoromethoxy) phenyl]hydrazinylidene]‐propanedinitrile (FCCP), complex I inhibitor rotenone, and complex III inhibitor Antimycin A. Intact cell respiration, oxidative phosphorylation, proton leak, maximum respiration (Max.R.), and reserve respiratory capacity (R.R.). (HC, *n* = 12; HD, *n* = 6; AH, *n* = 12). (B) Intact cell responses in a validation cohort (HC, *n* = 7; AH, *n* = 10). (C) Oxygen consumption measured in permeabilised PBMC in the same validation cohort. Responses to Malate (M), Pyruvate (P), ADP (D), Glutamate (G), and Succinate (S), Max.R., Complex II, R.R., rotenone‐insensitive/sensitive, and Complex IV respiration. Mean ± SD *p < .05; **p < .01; ***p *<* .001. AH Alcohol‐associated hepatitis; HC healthy control; HD heavy drinkers with no liver disease.

Functional studies were done in a two‐step manner. In the discovery cohort, studies in intact PBMC from AH, HC, and HD were performed using the CPT protocol in fresh cells. These studies were then replicated in a separate, independent, validation cohort of AH and HC in intact PBMC. Biochemical mechanistic studies were then performed in permeabilised PBMC. In the *discovery cohort*, intact PBMC from AH (*n* = 12) had significantly lower (*p* < .05 or lower) intact cell, ATP‐linked, maximum respiration, and reserve respiratory capacity than HC (*n* = 12) while responses in HD (*n* = 6) were similar to those in AH (Figure 3A). These data showed impaired oxidative phosphorylation in intact, fresh PBMC from patients with AH.

Mitochondrial oxidative function was then measured in both intact and permeabilised cells in a separate validation cohort. Responses of intact PBMC (ATP‐linked, maximum respiration, reserve respiratory capacity) were similar to those in our discovery cohort (Figure 3B). We then dissected the biochemical mechanisms of mitochondrial oxidative dysfunction in PBMC. Since substrates for ETC components do not enter intact cells, SUIT studies in permeabilised PBMCs from the same patients with AH and HC as in the validation cohort were performed concurrently with the intact cell studies. Consistently, PBMC from patients with AH had lower oxygen consumption compared to HC in response to complex I substrates (malate, pyruvate, glutamate), and succinate, a complex II substrate. Maximum respiration measured by titration of response to uncoupler FCCP, and responses to rotenone (complex I inhibitor) were also lower in PBMC from AH patients compared to those from HC. Thus, complex I‐ and II‐, but not complex IV‐dependent respiration was lower in PBMC from AH (Figure 3C). Our transcriptomic data showed differential alteration (increase/decrease) of mitochondrial ETC genes in a cell type–specific manner while our functional studies in the global PBMC pool isolated using protocols optimised for mitochondrial function showed lower activity in situ for complex I and II.

Mitochondrial oxidative dysfunction is associated with alterations in TCA cycle intermediates.^25^ Also, our scRNAseq and integrated pseudobulk analyses showed significant increase in transcripts, primarily in monocytes, in genes for enzymes in the TCA cycle. Again, transcripts do not necessarily correlate with functional responses, and may be higher as an adaptive mechanism when there is lower functional activity. We therefore measured the concentrations of intermediary metabolites in plasma and PBMC in the various groups of subjects.

### 3.4 Intermediary metabolite concentrations

**FIGURE 4:**
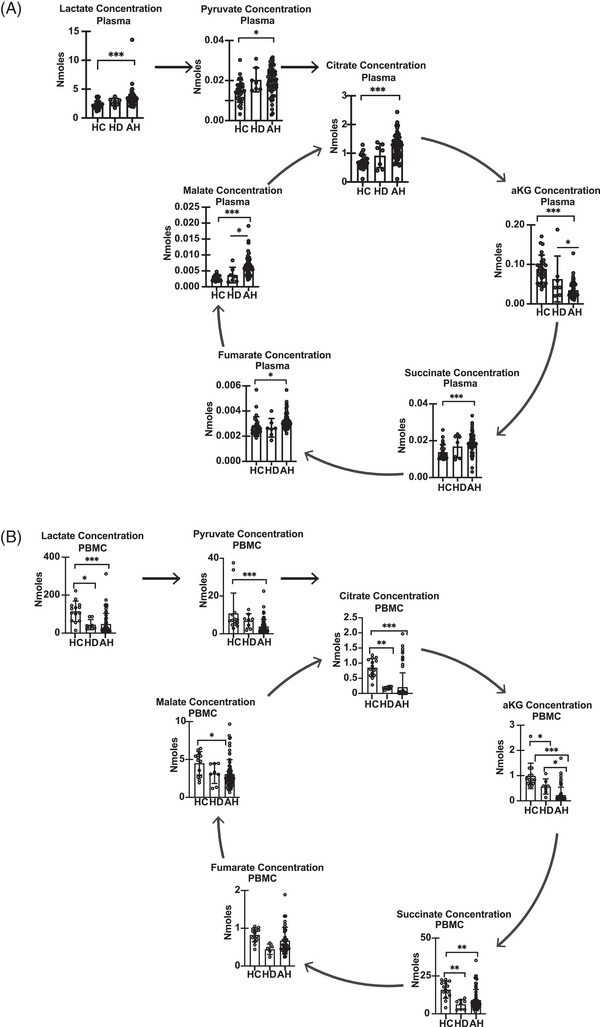
Altered circulating intermediary metabolites in patients with alcohol‐associated hepatitis. (A) Plasma tricarboxylic acid (TCA) cycle intermediates lactate, pyruvate, citrate, alpha‐keto glutarate, succinate, fumarate, and malate (HC = 27, HD = 8, AH = 59). (B) TCA cycle intermediates lactate, pyruvate, citrate, alpha‐keto glutarate (αKG), succinate, fumarate, and malate in peripheral blood mononuclear cells (PBMC). *p *<* .05; ***p *<* .001. All data expressed as mean ± SD (HC = 18; HD = 8; AH = 69). AH Alcohol‐associated hepatitis; HC healthy control; HD heavy drinkers with no liver disease.

Plasma concentrations of most intermediary metabolites (Figure 4A) were higher (*p* < .05 or lower) in AH (*n* = 59) than in HC (*n* = 27). Plasma concentrations of lactate, pyruvate, citrate, succinate, and fumarate were similar in AH and HD (*n* = 8). Interestingly, plasma concentrations of α‐ketoglutarate were lower and malate werehigher in AH than that in HC or HD. There were no significant differences in plasma concentrations of intermediary metabolites between HC and HD. Intermediary metabolites were quantified in PBMC from AH (*n* = 69), HD (*n* = 8) and HC (*n* = 14) that showed that all intermediary metabolites except fumarate were lower in AH than in HC. Similar to plasma concentrations, α‐ketoglutarate was lower in AH than in HC or HD. Intermediary metabolites lactate, citrate, α‐ketoglutarate, and succinate were lower in HD than HC, while pyruvate, fumarate, and malate concentrations were similar in HD and HC (Figure 4B). Distinct segregation of intermediary metabolite concentrations in PBMC, but not plasma, was noted in patients with spontaneous bacterial peritonitis (higher concentrations) and higher numbers of hospitalisations (lower concentrations) suggesting a relation between clinical findings on these metabolites (Table S24).

### 3.5 Senescence in PBMC in AH

**FIGURE 5:**
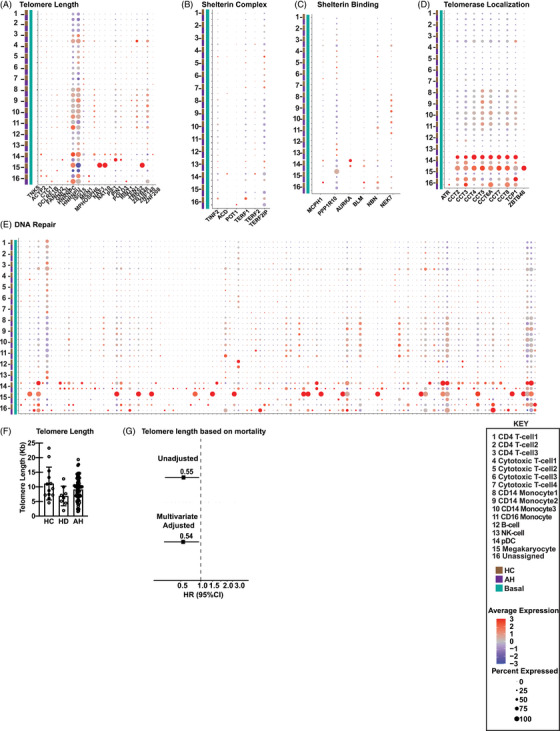
Peripheral blood mononuclear cell senescence in alcohol‐associated hepatitis. Feature extraction of mitochondrial genes was followed by Uniform Manifold Approximation and Projection (UMAP) and Seurat analysis to identify clustering by specific cell type based on mitochondrial gene expression in peripheral blood mononuclear cells (PBMC) from control (HC) or alcohol‐associated hepatitis (AH) subjects at baseline (*n* = 4 each). Schematic dot‐plot representation of single‐cell RNA sequencing (scRNAseq) data of comparative average expression for (A) Telomere Length; (B) Shelterin complex; (C) Shelterin‐binding genes; (D) Telomerase localisation and (E) DNA damage repair. (F) Experimental validation of telomere length in PBMC by real‐time PCR in HC (*n* = 13), AH (*n* = 72) and heavy drinkers (HD) (*n* = 8). (G) Hazard ratio of mortality based on telomere length in AH (*n* = 72).

Telomere shortening is a measure of cellular senescence.^35^ Dot plots that allow for simultaneous comparisons of expression and percentage of cells expressing genes on scRNAseq were generated for telomere regulatory pathways (Table S25).^27^ Cell type– and group‐specific (HC or AH in the basal unstimulated state) patterns of differential expression of genes related to telomere length, telomeric repeat‐containing RNA (TERRA), alternative lengthening of telomeres (ALT) repressing genes, shelterin‐binding, telomere repeat binding, DNA‐repair, telomerase activity, and telomerase localisation were noted (Figures 5A–E and S11A–F; Tables S26 and 27). Monocytes and pDC again showed the greatest change in expression patterns with a consistent pattern of an increase in telomere protection/DNA damage repair pathway genes in PBMC from AH compared to controls.

To experimentally validate these bioinformatic analyses, telomere length was measured in AH (*n* = 72), HC (*n* = 13), and HD (*n* = 8). Telomere length as a ratio of a known genomic reference (T/S) as well as log‐transformed values showed no differences in the various subject groups (Figure 5F). We then Z‐score adjusted the telomere length as previously described^36^ to minimise the impact of batch effect across multiple samples, and Youden optimum cut points were calculated^37^ based on sensitivity and specificity of telomere length with mortality as the dependent variable. Lower telomere length in patients with AH was associated with higher mortality (Figure 5G).

Consistent with mitochondrial oxidative dysfunction being associated with accelerated senescence, patients with AH who had elevated (> 1.5 SD over mean) intermediary metabolite in PBMC had higher telomere length but not mitochondrial oxidative function or survival (Table S24).

## 4 DISCUSSION

There is increasing interest in the role of PBMC function in immune cell responses and as a biomarker for disease severity, prognosis and therapeutic response. Our bioinformatics‐functional studies showed that on scRNAseq, mitochondrial regulatory genes were significantly different between AH and HC. We then integrated the scRNAseq data to generate the global responses in PBMC and showed mitochondrial oxidative function, TCA cycle regulation, free radical generation, and antioxidant genes were significantly altered in AH. Processing methods and freeze‐thaw affected mitochondrial functional responses, as evidenced by consistently lower mitochondrial oxidative function in PBMC separated by Ficoll gradient rather than CPT‐based separation in AH and HC subjects. Thawed PBMC had lower mitochondrial oxidative functional responses than freshly isolated PBMC from the same AH and HC subjects. Simultaneous measurements of mitochondrial oxidative function in freshly isolated PBMC using the CPT protocol in intact and permeabilised cells showed lower complex I and II function in AH compared to HC or HD. Circulating levels of intermediary metabolites in plasma were higher in AH than HC, while those in PBMC were lower. Finally, telomere length, a measure of replicative senescence, was lower in PBMC from patients with AH who died compared to those who survived. The clinical, laboratory findings, and outcomes in patients with AH are similar to those previously reported.^38^ In the present studies, we used fresh PBMC from consecutive patients to allow for functional studies to complement biochemical analyses in frozen cells. Mortality in the AH group was expectedly high given the high MELD scores at inclusion while none of the HC or HD died during the follow‐up.

Unbiased RNAseq data generated in PBMC from AH and HC showed differences in expression of genes regulating mitochondrial functional responses including oxidative phosphorylation, TCA cycle, FR generation, and antioxidant genes in a cell type–specific manner on scRNAseq and cell agnostic manner on integrated pseudobulk RNAseq. Cellular senescence is a consequence of mitochondrial oxidative dysfunction and ethanol exposure and AH result in increased senescence markers in different cell types.^39^, ^40^, ^41^ We report enrichment of senescence pathways including genes regulating telomere length on scRNAseq from PBMC in AH compared to HC and HD, suggesting immune senescence. The most changes were noted in pDC despite their constituting only a small percentage of the PBMC pool. Since pDC can detect pathogen‐derived nucleic acids and respond with rapid production of type 1 interferon, these cells need the bioenergetic plasticity provided by mitochondrial oxidative function,^42^ which could explain the high mitochondrial gene changes in this subtype of PBMC. Global or bulk RNAseq would have yielded the results obtained on integrated pseudobulk analyses done by us, but would not allow for identifying the individual cell type–based responses noted on scRNAseq. Such an analysis lays the foundation for future single‐cell functional responses by approaches including amplification of single‐cell populations or enhanced sensitivity of assays for functional studies. Integration of scRNAseq data has the potential to result in overcontribution by some cell types which can alter the significance of other cell types. However, while our functional studies reflect integrated gene expression rather than individual cell functions, our approach of using scRNAseq data reveals the contributions of individual cell types also.

A number of methods have been used to process PBMC for functional and unbiased data analyses.^14^, ^43^, ^44^ We show that paired studies in PBMC isolated from the same subject had higher oxidative responses with CPT than Ficoll density gradient. These data are consistent with reports by others who evaluated cell recovery, viability, frequency of immune cell subsets or T‐cell function in PBMC isolated using CPT or a similar protocol using Lymphoprep.^43^ Interestingly, both CPT and Lymphoprep use similar principles of a barrier with density gradient rather than the standard Ficoll‐Paque HIstopaque®−1077 approaches. However, when using the CPT for PBMC isolation, viability was similar in frozen and fresh cells. Cytokine production was also similar between fresh and frozen PBMC,^43^ but these are not measures of mitochondrial oxidative function quantified in the present studies. Our observations are also consistent with reports in PBMC from patients with HIV and healthy controls that showed consistent changes in cytokine responses.^29^ The type of additive used during cryopreservation also significantly affected functional responses, but foetal bovine serum used in our protocol does not affect viability or cytokine responses during preservation.^30^ Our data suggest that using a barrier containing tube for separation of PBMC is efficient and allows for maintained mitochondrial oxidative function compared to standard density gradient centrifugation protocols. Even though there are very limited data on the impact of freezing on functional responses, there is a hierarchy of energy utilisation of cellular processes that may contribute to context‐specific differences between fresh and frozen cells, especially in disease states. Therefore, the use of fresh PBMC rather than frozen cells is a preferred method to determine PBMC functional responses in AH.

Our observations in fresh PBMC isolated using the CPT protocol are consistent with previous data that patients with AH have mitochondrial oxidative dysfunction in liver and skeletal muscle.^8^, ^9^, ^10^ Previous reports on transcriptomics of PBMC have suggested mitochondrial dysfunction.^17^, ^20^ In the present studies, we show that functional defects occur in complex I and II in the ETC. Ethanol differentially decreased the expression of a number of the P‐, N‐ and Q‐modules of complex I of the ETC and multiple components of other complexes. To dissect the specific defects in ETC components, studies were performed in digitonin permeabilised cells since substrates for the complexes do not permeate the plasma membrane.^45^ Consistent with our scRNAseq data analyses, we observed lower complex I function in PBMC in AH compared to HC. Interestingly, complex II (succinate dehydrogenase) function was also lower in AH than HC suggesting that major mechanisms of substrate oxidation are impaired with AH and may explain the higher plasma TCA cycle intermediate concentrations (succinate is a critical metabolite in the TCA cycle). Impaired complex I and II function in PBMC in patients with AH is consistent with our observations that ATP‐linked respiration in intact cells was lower in AH. In addition to defects in components of the ETC, plasma and PBMC concentrations of TCA cycle intermediary metabolites also are altered in AH and contribute to mitochondrial oxidative responses. Our data from targeted quantification that showed higher plasma TCA cycle intermediary metabolites were similar to previous reports on untargeted metabolomics in patients with AH and targeted metabolomics in patients with nonalcoholic fatty liver disease.^31^, ^46^ Interestingly, we noted lower concentrations of TCA cycle intermediary metabolites in PBMC from AH compared to HC. It is not clear if these responses are mechanistically linked to mitochondrial oxidative dysfunction in PBMC in AH. It is possible that lower mitochondrial oxidative responses initiate adaptive responses with anaplerosis that results in higher concentrations of intermediary metabolites that leak from cells but the potential diagnostic and prognostic relevance of these observations need to be evaluated in future. Since plasma concentrations can be derived from multiple tissue sources, it is also possible that the discord between plasma and PBMC concentrations may be due to lower uptake rather than leak of metabolites and studies on the mechanistic and metabolic basis of these changes need to be evaluated in future.

Mitochondrial dysfunction contributes to cellular and immune senescence.^47^, ^48^, ^49^ Our bioinformatics analyses showed enrichment in senescence pathways specifically telomere maintenance. Interestingly, our experimental data showed no difference in telomere length in patients with AH compared with HC and HD. However, lower telomere length based on Z‐score cutoff was related to mortality in patients with AH. There is conflicting published data on the relation between alcohol consumption and telomere length.^50^ Interestingly, a Mendelian randomisation study showed that a diagnosis of alcohol use disorder rather than the number of drinks was associated with lower telomere length.^51^ Our integrated bioinformatics‐experimental analyses suggest potential mechanistic bases for lower telomere length because many of the telomere maintaining molecules in different pathways including shelterin, and telomerase (Table S25) on scRNAseq are potential molecular contributors to lower telomere length in nonsurvivors in the AH group. There were no significant differences in unadjusted telomere length between the different groups of subjects, which may be due to batch effects. Using standardised Z‐score of log‐transformed telomere length to minimise the impact of batch effects and using Youden's cut‐point criteria,^32^, ^33^ we showed that lower telomere length in AH was associated with higher mortality. Our approach to use standardised scores with optimal cut points has been used by others to assess the strength of biomarkers^37^ and telomere length is increasingly considered to be a biomarker of ‘biological age.’^35^ One possible reason for the discord between the integrated pseudobulk scRNAseq and experimental data on telomere length may be due to the heterogeneity in telomere regulatory genes in subpopulations of PBMC and our experimental approach of quantifying telomere length in the whole PBMC population. Also, the telomere regulatory genes including those involved in multiple processes show heterogeneity on scRNAseq even within the same cell type, but the overall effect is the consequence of interaction of multiple regulatory molecules rather than one or two specific telomere regulatory genes. For instance, shelterins protect the telomere length, but there are different components of the shelterin complex and dissecting the details will require newer approaches to amplify individual cell fractions. Our data do show, however, that telomere length alterations occur in conjunction with and not independent of the gene changes in PBMC. Future studies on telomere length measurements in subpopulations of PBMC may provide insights into differential replicative senescence in response to alcohol or in AH. Lack of complete concordance between integrated pseudobulk transcriptomics and experimental observations is similar to that previously reported in other model systems also.^52^ Moreover, our experimental data suggests accelerated senescence as evidenced by shorter telomere length in the subpopulation of AH with a high mortality. These findings show the need for future studies and modelling which incorporate telomere length to predict clinical outcomes in the AH population.

Our complementary bioinformatics‐based approaches and validation of mitochondrial oxidative functional responses show that even though frozen cells can be used for unbiased and metabolite studies, fresh PBMC processed using CPT protocols provide consistent mitochondrial oxidation responses in both intact and permeabilised cells. In patients with AH, consistent with our integrated pseudobulk RNAseq analyses, lower complex I and II function were noted with no evidence of telomere shortening in the whole PBMC population despite changes in individual cell subpopulations on single‐cell transcriptomics (Graphical abstract). These data provide support for standardised protocols for PBMC isolation, evaluate functional responses in individual cell types, and provide the basis for quantifying circulating intermediary metabolites as potential biomarkers for outcomes in AH.

## AUTHOR CONTRIBUTIONS

Designing studies: SD, AB, JD, NW. Conduct experiments: AB, JD, NW, AA, JS, SM, AK, RS, YS. Acquiring data: AB, JD, NW, AA, JS, SM, AK, RM, RS, YS. Writing manuscript: AB, NW, JD, AA, RM, AK, JS, SM, RS, YS, DS, LEN, SD.

## CONFLICT OF INTEREST STATEMENT

The authors declare no conflicts of interest.

## Supporting information

Supplementary information

Supplementary information

Supplementary information

Supplementary information

Supplementary information

Supplementary information

Supplementary information

Supplementary information

Supplementary information

Supplementary information

Supplementary information

Supplementary information

Supplementary information

Supplementary information

Supplementary information

Supplementary information

Supplementary information

Supplementary information

Supplementary information

Supplementary information

Supplementary information

Supplementary information

Supplementary information

Supplementary information

Supplementary information

Supplementary information
