# Supplementary material for: Peripheral blood mononuclear cell mitochondrial dysfunction in acute alcohol‐associated hepatitis
Source: Clin Transl Med. 2023 May 25;13(5):e1276. doi: 10.1002/ctm2.1276 (PMC10212276; doi:10.1002/ctm2.1276)
Supplement: Supplementary file 1 — Supplementary information [file CTM2-13-e1276-s012.docx]

Index of Supplementary Tables

**Supplementary Table 1**. Number of patients for each experiment

**Supplementary Table 2**. Data availability

**Supplementary Table 3**. Gene list of all MitoCarta/mitochondrial genes (supervised)

**Supplementary Table 4**. Gene list of all ETC genes (supervised)

**Supplementary Table 5**. Gene list of all OXPHOS genes (supervised)

**Supplementary Table 6**. Gene list of all MitoCarta/mitochondrial genes (unsupervised)

**Supplementary Table 7**. Gene list of all OXPHOS genes (unsupervised)

**Supplementary Table 8**. Gene list of all TCA genes (supervised)

**Supplementary Table 9**. Gene list of all glycolysis genes (supervised)

**Supplementary Table 10**. Gene list of all ROS genes (supervised)

**Supplementary Table 11**.Gene list of all antioxidant genes (supervised)

**Supplementary Table 12**. Patient details of cell processing Ficoll vs Cell Processing Tube (CPT)

**Supplementary Table 13**. Patient details of fresh vs frozen cells analysis

**Supplementary Table 14**. Patient details of single cell RNA sequencing

**Supplementary Table 15**. Patient details for the discovery cohort

**Supplementary Table 16**. Patient details for the validation cohort (intact cell respiration)

**Supplementary Table 17**. Patient details for the validation cohort (permeabilized cells)

**Supplementary Table 18**. Patient details for telomere length data

**Supplementary Table 19**. Patient details for plasma tricarboxylic acid cycle intermediate concentrations

**Supplementary Table 20**. Patient details for TCA cycle intermediate concentrations in PBMC

**Supplementary Table 21**. Patient details for TCA cycle intermediate concentrations in PBMC (subanalysis of alcohol associated hepatitis only)

**Supplementary Table 22**. Patient details for fatigue data (from PROMIS questionnaire)

**Supplementary Table 23**. Survivors vs Non-Survivors in alcohol associated hepatitis

**Supplementary Table 24**. Clinical and laboratory findings in patients with elevated intermediary metabolites in peripheral blood mononuclear cells from patients with alcohol associated hepatitis

**Supplementary Table 25**. Genes involved in telomere length maintenance.

**Supplementary Table 26**. Gene list of all DNA repair Genes

**Supplementary Table 27**. Gene list of all ALT Enhancing Genes

**Abbreviations:** ALT: Alternative Lengthening of Telomeres; CPT: Cell Processing Tube, ETC: electron Transport Chain, OXPHOS: oxidative phosphorylation, PBMC: Peripheral Blood Mononuclear Cells; PROMIS: Patient Reported Outcomes Measurement Information System, ROS Reactive Oxygen Species, TCA: tricarboxylic acid.
