# Supplementary material for: Peripheral blood mononuclear cell mitochondrial dysfunction in acute alcohol‐associated hepatitis
Source: Clin Transl Med. 2023 May 25;13(5):e1276. doi: 10.1002/ctm2.1276 (PMC10212276; doi:10.1002/ctm2.1276)
Supplement: Supplementary file 13 — Supplementary information [file CTM2-13-e1276-s005.docx]

**Supplementary Figure Legends**

**S.Fig.1. UpSet plots showing subjects in various groups**. Shared and unique subjects across different analyses. Rows are the different experimental sets and columns are the intersections (subjects shared between experimental sets). Numbers on columns and rows show number of subjects. CPT cell processing tube; PBMC peripheral blood mononuclear cells; TCA tricarboxylic acid cycle.

**S. Fig. 2. Single cell RNA sequencing expression data of electron transport chain complexes**. Uniform manifold approximation and projection (UMAP) graphs of genes of components of each of the mitochondrial complex genes in peripheral blood mononuclear cells from patients with alcohol associated hepatitis (AH) and healthy controls (HC) in the basal state and in response to lipopolysaccharide stimulation. Seurat analysis used for clustering of cell types. **A**. Complex 1 components MT-ND 1-4; MT-ND4L; MT-ND5; MT-ND6; MT-NDUFS 1-3,7,8; NDUFV 1,2; NDUFAB1,NDUF A1-3, 5-13; NDUFB1-11; NDUFC1,2; NDUFS4-6; NDUFV3. **B**. Complex II components SDH A-D. **C**. UQCRB; UQCRQ; UQCRC 1,2; MT-CYB; CYC1; UQCRFS1; UQCRH; UQCR10,11; **D**. COX4I1; COX5 A,B; COX6A1,E1,C; COX7A2, B,C; COX 8A; MT-CO1-3. **E**. ATP5F1 A-E; ATP5MC 1-3; ATP5M E-G; MT-ATP6,8; ATP5P E-F; ATP5PO; ATP5I F1. All data from n=4 in each group.

**S.Fig. 3**. **Heatmaps of mitochondrial genes in peripheral blood mononuclear cells**. Single cell RNA sequencing (scRNAseq) of peripheral blood mononuclear cells from patients with alcohol associated hepatitis (AH) and healthy controls (HC) in the basal state and in response to lipopolysaccharide stimulation **A,B**. Unsupervised heatmaps of mitochondrial genes and oxidative phosphorylation molecules in pseudobulk integrated scRNAseq data. **C**. Supervised heatmap of OXHPOS genes expressed in individual cell types of the PBMC population on scRNAseq. Cell types with unique patterns and increased expression in AH as compared to healthy controls include monocytes (CD-14 types 1-3 and CD-16) CD-4 type 3 cells, and pDCs. Additionally cytotoxic T cells types 1-3 show different patterns between the two groups. N= 4 in each group. OXPHOS Oxidative phosphorylation; PBMC peripheral blood mononuclear cells; pDC plasmacytoid dendritic cells.

**S. Fig. 4. Comparative expression of electron transport chain complex I components**. Schematic dot-plot representation of comparative average expression of components of the electron transport chain (ETC) complex I transcript expression in single cell RNA sequencing (scRNAseq) of peripheral blood mononuclear cells (PBMC) from patients with alcohol associated hepatitis (AH) an healthy controls (HC) in the basal state and in response to lipopolysaccharide (LPS) stimulation. Seurat analysis used for clustering of cell types on scRNAseq. **A**. AH and HC with LPS stimulation. **B**. HC in basal state and with LPS stimulation. **C**. AH in basal state and with LPS stimulation. Darker color represents higher and lighter color lower average expression. Size of dot represents percentage of cells expressing specified gene (smaller-lower percentage/larger- higher percentage of cells). All data from scRNAseq from n=4 in each group. Full Gene Names for Panel 1B: MT-ND1, MT-ND2, MT-ND3, MT-ND4, MT-ND4L, MT-ND5, MT-ND6, NDUFAB1, NDUFA1, NDUFA3, NDUFA8, NDUFA10, NDUFA11, NDUFA13, NDUFB1, NDUFB2, NDUFB3, NDUFB4, NDUFB5, NDUFB6, NDUF7, NDUFB8, NDUFB9, NDUFB10, NDUFB11, NDUFC1, NDUFC2, NDUFS5, NDUFS1, NDUFV1, NDUFV2, NDUFA2, NDUFA12, NDUFS4, NDUFS6, NDUFV3, NDUFS2, NDUFS3, NDUFS7, NDUFS8, NDUFA5, NDUFA6, NDUFA7, NDUFA9.

**S.Fig. 5**. **Comparative expression of electron transport chain complex II components**. Average expression of components of the electron transport chain (ETC) complex II transcripts in single cell RNA sequencing (scRNAseq) of peripheral blood mononuclear cells (PBMC) from patients with alcohol associated hepatitis (AH) and healthy controls (HC) in the basal state and in response to lipopolysaccharide (LPS) stimulation. **A**. Supervised heat map of single cell RNA sequencing in HC and AH in the basal state. **B-E**. Schematic dot-plot representation of comparative average transcript expression in the following groups: **B**. HC and AH in the basal state. **C**. HC and AH with LPS stimulation. **D**. HC in basal state and with LPS stimulation. **E**. AH in basal state and with LPS stimulation. Darker color represents higher and lighter color lower average expression. Size of dot represents percentage of cells expressing specified gene (smaller-lower percentage/larger- higher percentage of cells). **F**. Heatmap of (pseudo-bulk) transcriptomics of ETC complex II components was generated by consolidation of scRNAseq. Seurat analysis was used for clustering of cell types on scRNAseq. All data from scRNAseq from n=4 in each group.

**S.Fig. 6**. **Comparative expression of electron transport chain complex III components.** Average expression of components of the electron transport chain (ETC) complex III transcripts in single cell RNA sequencing (scRNAseq) of peripheral blood mononuclear cells (PBMC) from patients with alcohol associated hepatitis (AH) and healthy controls (HC) in the basal state and in response to lipopolysaccharide (LPS) stimulation. **A**. Supervised heat map of single cell RNA sequencing in HC and AH in the basal state. **B-E**. Schematic dot-plot representation of comparative average transcript expression in the following groups: **B**. HC and AH in the basal state. **C**. HC and AH with LPS stimulation. **D**. HC in basal state and with LPS stimulation. **E**. AH in basal state and with LPS stimulation. Darker color represents higher and lighter color lower average expression. Size of dot represents percentage of cells expressing specified gene (smaller-lower percentage/larger- higher percentage of cells). **F**. Heatmap of (pseudo-bulk) transcriptomics of electron transport chain complex III components was generated by consolidation of scRNAseq. Seurat analysis was used for clustering of cell types from scRNAseq. All data from scRNAseq from n=4 in each group.

**S.Fig. 7**. **Comparative expression of electron transport chain complex IV components.** Average expression of components of the electron transport chain (ETC) complex IV transcripts in single cell RNA sequencing (scRNAseq) of peripheral blood mononuclear cells (PBMC) from patients with alcohol associated hepatitis (AH) and healthy controls (HC) in the basal state and in response to lipopolysaccharide (LPS) stimulation. **A**. Supervised heat map of single cell RNA sequencing in HC and AH in the basal state. **B-E**. Schematic dot-plot representation of comparative average transcript expression in the following groups: **B**. HC and AH in the basal state. **C**. HC and AH with LPS stimulation. **D**. HC in basal state and with LPS stimulation. **E**. AH in basal state and with LPS stimulation. Darker color represents higher and lighter color lower average expression. Size of dot represents percentage of cells expressing specified gene (smaller-lower percentage/larger- higher percentage of cells). **F**. Heatmap of average expression on pseudo-bulk transcriptomics of electron transport chain complex IV components was generated by integration of scRNAseq. Seurat analysis was used for clustering of cell types from scRNAseq. All data from scRNAseq from n=4 in each group.

**S.Fig. 8**. **Comparative expression of electron transport chain complex V components.** Average expression of components of the electron transport chain (ETC) complex V transcripts in single cell RNA sequencing (scRNAseq) of peripheral blood mononuclear cells (PBMC) from patients with alcohol associated hepatitis (AH) and healthy controls (HC) in the basal state and in response to lipopolysaccharide (LPS) stimulation. **A**. Supervised heat map of scRNAseq in HC and AH in the basal state. **B-E**. Schematic dot-plot representation of comparative average transcript expression in the following groups: **B**. HC and AH in the basal state. **C**. HC and AH with LPS stimulation. **D**. HC in basal state and with LPS stimulation. **E**. AH in basal state and with LPS stimulation. Darker color represents higher and lighter color lower average expression. Size of dot represents percentage of cells expressing specified gene (smaller-lower percentage/larger- higher percentage of cells). **F**. Heatmap of (pseudo-bulk) transcriptomics of electron transport chain complex V components generated by consolidation of scRNAseq. Seurat analysis was used for clustering of cell types from scRNAseq. All data from scRNAseq from n=4 in each group.

**S.Fig. 9. Heatmaps of glycolysis, tricarboxylic acid cycle and free radical genes**. Expression data from single cell RNA sequencing (scRNAseq) of peripheral blood mononuclear cells (PBMC) from patients with alcohol associated hepatitis (AH) and healthy controls (HC) in the basal state and in response to lipopolysaccharide (LPS) stimulation. **A**. Average expression of glycolytic pathway enzyme genes on scRNAseq. **B-E**. Supervised heat map of average expression on pseudobulk transcriptomics generated by integration scRNAseq expression data for , **(B)** tricarboxylic acid (TCA) cycle, **(C)**glycolysis, **(D)** free radical, and **(E)** antioxidant genes. Seurat analysis was used for clustering of cell types from scRNAseq. All data from scRNAseq from n=4 in each group.

**S.Fig. 10. Isolation protocols affect mitochondrial oxidative function.** Comparisons of mitochondrial oxidative function responses in peripheral blood mononuclear cells (PBMC) from healthy controls and patients with alcohol associated hepatitis. Responses to oligomycin (ATP synthase inhibitor); FCCP (oxidative phosphorylation uncoupler); rotenone (complex I inhibitor) and antimycin A (AA; complex III inhibitor) were quantified. Intact cell respiration, phosphorylation dependent oxygen consumption, proton leak, maximum respiration, and reserve respiratory capacity. **A,B.** Intact PBMC responses in fresh cells from the same subject (11 controls and 4 AH) separated by Ficoll and cell preparation tube (CPT) protocols. **C,D.** Comparison of mitochondrial respiration in PBMC isolated using CPT in fresh cells that were studied immediately after isolation and cells that were frozen overnight and thawed the following morning for analyses. Fresh/frozen n = 16 (7 controls and 9 AH). All data were collected from PBMCs isolated using the CPT method. Data show individual reads and mean±standard deviation. **P <*0.05; ***P <*0.01.

**S.Fig. 11. Expression of telomere regulatory pathway genes.** Schematic dot-plot representation of comparative average expression of components of the telomere regulatory pathway gene transcripts in single cell RNA sequencing (scRNAseq) of peripheral blood mononuclear cells (PBMC) from patients with alcohol associated hepatitis (AH) and healthy controls (HC) in the basal state. **A**. Alternate lengthening of telomeres (ALT) enhancing. **B**. ALT repressing. **C**. Extrachromosomal repeat binding. **D**. Sumoylation. **E**. Telomeric Repeat containing RNA (TERRA). **F**. Telomere Repeat Binding. Seurat analysis was used for clustering of cell types from scRNAseq. All data from scRNAseq from n=4 in each group.
