# Supplementary material for: Peripheral blood mononuclear cell mitochondrial dysfunction in acute alcohol‐associated hepatitis
Source: Clin Transl Med. 2023 May 25;13(5):e1276. doi: 10.1002/ctm2.1276 (PMC10212276; doi:10.1002/ctm2.1276)
Supplement: Supplementary file 14 — Supplementary information [file CTM2-13-e1276-s006.docx]

**S.Table 1. Number of patients for each experiment**

|  | Number of Patients | | |
| --- | --- | --- | --- |
|  | **HC** | **HD** | **AH** |
| ScRNA | 4 | 0 | 4 |
| CPT vs Ficoll | 11 | 0 | 4 |
| Fresh vs Frozen | 6 | 0 | 10 |
| Discovery Cohort | 12 | 6 | 12 |
| Validation Cohort (Intact) | 7 | 0 | 10 |
| Validation Cohort (Protocol 1) | 7 | 0 | 10 |
| Telomere Length | 13 | 8 | 72 |
| TCA Plasma | 27 | 8 | 59 |
| TCA PBMC | 14 | 8 | 69 |
| TCA PBMC (subanalysis) | 0 | 0 | 69 |
| Fatigue Data | 17 | 3 | 12 |

AH acute alcohol associated liver disease; CPT cell processing tube; HC healthy controls; HD heavy drinkers without liver disease; PBMC peripheral blood mononuclear cells; scRNAseq single cell RNA sequencing; TCA tricarboxylic acid cycle;

**S.Table 2. Data availability**

|  | **HC** | **HD** | **AH** |
| --- | --- | --- | --- |
| Total number | 31 (100%) | 11 (100%) | 81 (100%) |
| Body mass index (kg/m^2^) | 29 (93.5%) | 11 (100% | 81 (100%) |
| Total leucocyte count (x1000/ml.) | 27 (87.1%) | 10 (90.9%) | 81 (100%) |
| Platelet (x1000/ml.) | 27 (87.1%) | 10 (90.9%) | 81 (100%) |
| Alanine amino transferase | 27 (87.1%) | 10 (90.9%) | 81 (100%) |
| Aspartate aminotransferase | 27 (87.1%) | 10 (90.9%) | 81 (100%) |
| Serum albumin | 27 (87.1%) | 10 (90.9%) | 81 (100%) |
| Serum total protein | 27 (87.1%) | 10 (90.9%) | 81 (100%) |
| Bilirubin | 27 (87.1%) | 10 (90.9%) | 81 (100%) |
| Alkaline phosphatase | 27 (87.1%) | 10 (90.9%) | 81 (100%) |
| Serum creatinine | 27 (87.1%) | 10 (90.9%) | 81 (100%) |
| Blood urea nitrogen | 27 (87.1%) | 10 (90.9%) | 81 (100%) |
| Serum sodium | 27 (87.1%) | 10 (90.9%) | 81 (100%) |
| International normalized ratio | 16 (51.6%) | 10 (90.9%) | 81 (100%) |
| Mean Survival | 31 (100%) | 11 (100%) | 81 (100%) |

Number of patients in each group (percentage)

AH acute alcohol associated liver disease; HC healthy controls; HD heavy drinkers without liver disease;
