# Supplementary material for: Peripheral blood mononuclear cell mitochondrial dysfunction in acute alcohol‐associated hepatitis
Source: Clin Transl Med. 2023 May 25;13(5):e1276. doi: 10.1002/ctm2.1276 (PMC10212276; doi:10.1002/ctm2.1276)
Supplement: Supplementary file 24 — Supplementary information [file CTM2-13-e1276-s019.docx]

|  | **HC** | **HD** | **AH** |
| --- | --- | --- | --- |
| Number | 11 |  | 4 |
| Male:Female | 5:6 |  | 1:3 |
| Age in years (mean + SD) | 39.00 + 14.32 |  | 48.75 + 15.20 |
| Alive:Dead | 11:0 |  | 2:2 |
| Race W:AA:A:U | 9:0:1:1 |  | 4:0:0:0 |
| Body mass index (kg/m^2^) | 25.30 + 6.11 |  | 26.37 + 3.21 |
| Total leucocyte count (x1000/ul.) | 6.31 + 1.59 |  | 9.63 + 2.00^**^ |
| Platelet (x1000/ul.) | 255.62 + 62.83 |  | 113.25 + 79.46^**^ |
| Alanine amino transferase (U/L) | 18.88 + 6.15 |  | 37.50 + 25.33 |
| Aspartate aminotransferase (U/L) | 21.25 + 6.04 |  | 100.75 + 51.34 ^***^ |
| Serum albumin (g/dL) | 4.53 + 0.34 |  | 2.48 + 0.52 ^***^ |
| Serum total protein (g/dL) | 7.34 + 0.36 |  | 5.63 + 0.45 ^***^ |
| Bilirubin (mg/dL) | 0.73 + 1.09 |  | 10.53 + 7.57 ^**^ |
| Alkaline phosphatase (U/L) | 74.25 + 27.02 |  | 137.75 + 85.91 |
| Serum creatinine (mg/dL) | 0.91 + 0.19 |  | 0.46 + 0.21^**^ |
| Blood urea nitrogen (mg/dL) | 11.75 + 2.38 |  | 12.75 + 13.67 |
| Serum sodium (mmol/L) | 140.0 + 2.07 |  | 136.50 + 4.20 |
| International normalized ratio | 0.98 + 0.45 |  | 1.93 + 0.46 ^**^ |
| Steroids (Y:N) | 0:11 |  | 1:3 |
| Antibiotics (Y:N) | 1:10 |  | 4:0 |
| Antibiotic Count | 1.4 + 4.5 |  | 14.2 + 9.0 |
| Tobacco Use  Yes:Quit:Never:Unknown | 0:1:3:7 |  | 2:0:2:0 |
| Liver Specific Outcomes | | | |
| LOS in days |  |  | 18.75 + 19.35  (4-46) |
| GI Bleed (Y:N) |  |  | 1:3 |
| Ascites (Y:N) |  |  | 3:1 |
| Average Number of Hospitalizations (Mean + SD) (Range) |  |  | 3.75 + 4.19  (1-10) |
| Hepatic Encephalopathy (Y:N) |  |  | 3:1 |
| Cirrhosis (Y:N) |  |  | 0:4 |
| Mean Survival From Enrollment in Days  (Mean + SD) (Range) |  |  | 566.25 + 583.05  (41-1114) |
| MELD Score  (Mean + SD) (Range) |  |  | 20.88 + 5.02  (14-25) |
| AFP (>3:<3:Not Done) |  |  | 2:0:2 |
| UTI (Y:N) |  |  | 0:4 |
| Sepsis (Y:N) |  |  | 0:4 |
| Pneumonia (Y:N) |  |  | 0:4 |
| SBP (Y:N) |  |  | 1:3 |

**S.Table 12. Patient Details of Cell Processing Ficoll vs Cell Processing Tube (CPT)**

****** p<0.01; *** p<0.001.

**Abbreviations:** **A:** Asian, **AA:** African American, **AFP:** alpha fetoprotein, **AH:** alcohol associated hepatitis, **dL:** deciliter, **g:** grams, **GI:** gastrointestinal; **HC:** healthy control, **HD:** healthy heavy drinker, **kg:** kilograms, **LOS:** Length of Stay, **m:** meters, **MELD:** model for end stage liver disease, **mg:** milligrams, **mmol:** millimole; **N:** No, **SBP:** spontaneous bacterial peritonitis, **SD:** Standard Deviation, **U:** Unknown, **U/L:** units per liter, **uL:** microliter, **UTI:** urinary tract infection, **W:** White, **Y:** Yes

**S.Table 13. Patient details of Fresh vs Frozen cells analysis**

|  | **HC** | **HD** | **AH** |
| --- | --- | --- | --- |
| Number | 6 |  | 10 |
| Male:Female | 3:3 |  | 3:7 |
| Age in years (mean + SD) | 48.33+ 10.23 |  | 46.10 + 10.85 |
| Alive:Dead | 6:0 |  | 6:4 |
| Race W:AA:A:U | 5:0:0:1 |  | 9:0:1:0 |
| Body mass index (kg/m^2^) | 23.45 + 5.68 |  | 27.00 + 4.64 |
| Total leucocyte count (x1000/ul.) | 5.22 + 1.98 |  | 12.13 + 5.27^*^ |
| Platelet (x1000/ul.) | 240.20 + 67.74 |  | 118.30 + 50.71^**^ |
| Alanine amino transferase (U/L) | 19.40 + 10.64 |  | 33.30 + 19.05 |
| Aspartate aminotransferase (U/L) | 26.00 + 15.68 |  | 97.30 + 58.08^*^ |
| Serum albumin (g/dL) | 4.34 + 0.21 |  | 2.83 + 0.66^***^ |
| Serum total protein (g/dL) | 7.30 + 0.38 |  | 6.13 + 0.96^*^ |
| Bilirubin (mg/dL) | 0.98 + 1.36 |  | 14.42 + 7.04^***^ |
| Alkaline phosphatase (U/L) | 75.60 + 13.01 |  | 149.30 + 85.51 |
| Serum creatinine (mg/dL) | 0.93 + 0.14 |  | 1.18 + 1.11 |
| Blood urea nitrogen (mg/dL) | 13.20 + 5.07 |  | 17.60 + 13.58 |
| Serum sodium (mmol/L) | 139.80 + 1.48 |  | 133.40 + 4.70^**^ |
| International normalized ratio | 1.00 + 0.00 |  | 1.92 + 0.42^***^ |
| Steroids (Y:N) | 0:6 |  | 5:5 |
| Antibiotics (Y:N) | 2:4 |  | 10:0 |
| Antibiotics Count | 2.7 + 6.0 |  | 24.7 + 21.8 |
| Tobacco Use  Yes:Quit:Never:Unknown | 0:0:4:2 |  | 5:2:3:0 |
| Liver Specific Outcomes | | | |
| LOS in days |  |  | 22 + 17 |
| GI Bleed (Y:N) |  |  | 4:6 |
| Ascites (Y:N) |  |  | 9:1 |
| Average Number of Hospitalizations (Mean + SD) (Range) |  |  | 4.00 + 3.77  (0-10) |
| Hepatic Encephalopathy (Y:N) |  |  | 8:2 |
| Cirrhosis (Y:N) |  |  | 5:5 |
| Mean Survival From Enrollment in Days  (Mean + SD) (Range) |  |  | 495.10 + 437.09  (41-1148) |
| MELD Score  (Mean + SD) (Range) |  |  | 25.30 + 5.67  (14-35) |
| AFP (>3:<3:Not Done) |  |  | 5:0:5 |
| UTI (Y:N) |  |  | 0:10 |
| Sepsis (Y:N) |  |  | 1:9 |
| Pneumonia (Y:N) |  |  | 2:8 |
| SBP (Y:N) |  |  | 6:4 |

*p<0.05; ****** p<0.01; *** p<0.001.

**Abbreviations:** **A:** Asian, **AA:** African American, **AFP:** alpha fetoprotein, **AH:** alcohol associated hepatitis, **dL:** deciliter, **g:** grams, **GI:** gastrointestinal; **HC:** healthy control, **HD:** healthy heavy drinker, **kg:** kilograms, **LOS:** Length of Stay, **m:** meters, **MELD:** model for end stage liver disease, **mg:** milligrams, **mmol:** millimole; **N:** No, **SBP:** spontaneous bacterial peritonitis, **SD:** Standard Deviation, **U:** Unknown, **U/L:** units per liter, **uL:** microliter, **UTI:** urinary tract infection, **W:** White, **Y:** Yes

**S.Table 14. Patient details of Single Cell RNA Sequencing**

|  | **HC** | **HD** | **AH** |
| --- | --- | --- | --- |
| Number | 4 |  | 4 |
| Male:Female | 4:0 |  | 4:0 |
| Age in years (mean + SD) | 43.25 + 16.92 |  | 46.25 + 17.29 |
| Alive:Dead | 4:0 |  | 2:2 |
| Race W:AA:A:U | 4:0:0:0 |  | 4:0:0:0 |
| Body mass index (kg/m^2^) | 26.80 + 6.27 |  | 27.17 + 4.14 |
| Total leucocyte count (x1000/ul.) | 5.66 + 1.80 |  | 12.47 + 6.09 |
| Platelet (x1000/ul.) | 236.75 + 81.84 |  | 167.00 + 52.59 |
| Alanine amino transferase (U/L) | 19.75 + 6.24 |  | 45.50 + 37.44 |
| Aspartate aminotransferase (U/L) | 23.00 + 8.12 |  | 63.75 + 43.46 |
| Serum albumin (g/dL) | 4.55 + 0.51 |  | 3.18 + 0.17^**^ |
| Serum total protein (g/dL) | 7.28 + 0.36 |  | 5.50 + 0.68^**^ |
| Bilirubin (mg/dL) | 1.20 + 1.48 |  | 16.40 + 13.15 |
| Alkaline phosphatase (U/L) | 70.25 + 4.79 |  | 116.25 + 38.40 |
| Serum creatinine (mg/dL) | 0.87 + 0.15 |  | 2.13 + 1.04^*^ |
| Blood urea nitrogen (mg/dL) | 11.25 + 2.06 |  | 36.50 + 22.13 |
| Serum sodium (mmol/L) | 140.75 + 1.50 |  | 134.50 + 4.04^*^ |
| International normalized ratio | 0.97 + 0.06 |  | 2.30 + 1.63 |
| Steroids (Y:N) | 0:4 |  | 3:1 |
| Antibiotics (Y:N) | 0:4 |  | 3:1 |
| Antibiotics Count | 0 |  | 20 + 23 |
| Tobacco Use  Yes:Quit:Never:Unknown | 0:0:2:2 |  | 0:2:1:1 |
| Liver Specific Outcomes | | | |
| LOS in days  (Mean + SD) |  |  | 16 + 18 |
| GI Bleed (Y:N) |  |  | 4:0 |
| Ascites (Y:N) |  |  | 3:1 |
| Average Number of Hospitalizations  (Mean + SD) (Range) |  |  | 3.25 + 3.30  (1-8) |
| Hepatic Encephalopathy (Y:N) |  |  | 4:0 |
| Cirrhosis (Y:N) |  |  | 2:2 |
| Mean Survival From Enrollment in Days  (Mean + SD) (Range) |  |  | 1024.00 + 844.91  (87-1755) |
| MELD Score  (Mean + SD) (Range) |  |  | 28.98 + 6.76  (21-35) |
| AFP (>3:<3:Not Done) |  |  | 3:0:1 |
| UTI (Y:N) |  |  | 0:4 |
| Sepsis (Y:N) |  |  | 0:4 |
| Pneumonia (Y:N) |  |  | 1:3 |
| SBP (Y:N) |  |  | 2:2 |

*p<0.05; ****** p<0.01.

**Abbreviations:** **A:** Asian, **AA:** African American, **AFP:** alpha fetoprotein, **AH:** alcohol associated hepatitis, **dL:** deciliter, **g:** grams, **GI:** gastrointestinal; **HC:** healthy control, **HD:** healthy heavy drinker, **kg:** kilograms, **LOS:** Length of Stay, **m:** meters, **MELD:** model for end stage liver disease, **mg:** milligrams, **mmol:** millimole; **N:** No, **RNA:** ribonucleic acid, **SBP:** spontaneous bacterial peritonitis, **SD:** Standard Deviation, **U:** Unknown, **U/L:** units per liter, **uL:** microliter, **UTI:** urinary tract infection, **W:** White, **Y:** Yes

**S.Table 15. Patient details for the Discovery Cohort**

|  | **HC** | **HD** | **AH** |
| --- | --- | --- | --- |
| Number | 12 | 6 | 12 |
| Male:Female | 6:6 | 4:2 | 5:7 |
| Age in years (mean + SD) | 38.75 + 13.68 | 41.50 + 14.57 | 50.00 + 13.11 |
| Alive:Dead | 12:0 | 6:0 | 7:5 |
| Race W:AA:A:U | 9:0:1:2 | 5:0:0:1 | 10:1:1:0 |
| Body mass index (kg/m^2^) | 25.12 + 5.83 | 28.84 + 6.84 | 28.16 + 5.11 |
| Total leucocyte count (x1000/ul.) | 6.09 + 1.62 | 7.11 + 3.14 | 13.17 + 8.00^a^ |
| Platelet (x1000/ul.) | 240.33 + 74.56 | 261.60 + 85.49 | 115.75 + 50.25^b^ |
| Alanine amino transferase (U/L) | 20.89 + 8.34 | 37.40 + 21.55 | 43.00 + 27.73 |
| Aspartate aminotransferase (U/L) | 21.44 + 5.68 | 26.40 + 4.22 | 94.92 + 45.27 ^b^ |
| Serum albumin (g/dL) | 4.54 + 0.32 | 4.40 + 0.19 | 2.77 + 0.64 ^b^ |
| Serum total protein (g/dL) | 7.37 + 0.35 | 7.28 + 0.39 | 6.03 + 0.94^c,d^ |
| Bilirubin (mg/dL) | 0.69 + 1.03 | 0.42 + 0.11 | 11.39 + 8.16^c,d^ |
| Alkaline phosphatase (U/L) | 73.00 + 25.55 | 83.80 + 21.15 | 165.67 + 90.82^e^ |
| Serum creatinine (mg/dL) | 0.91 + 0.18 | 0.80 + 0.07 | 0.98 + 0.80 |
| Blood urea nitrogen (mg/dL) | 12.56 + 3.28 | 12.40 + 3.58 | 18.75 + 14.24 |
| Serum sodium (mmol/L) | 140.00 + 1.94 | 138.20 + 3.11 | 133.92 + 4.42^e^ |
| International normalized ratio | 1.03 + 0.14 | 1.00 + 0.07 | 1.88 + 0.42 ^b^ |
| Steroids (Y:N) | 0:12 | 0:6 | 7:5 |
| Antibiotics (Y:N) | 2:10 | 1:5 | 10:2 |
| Antibiotics Count | 1.3 + 4.33 | 2.8 + 6.9 | 20.3 + 22.2 |
| Tobacco Use  Yes:Quit:Never:Unknown | 0:1:4:7 | 1:0:1:4 | 5:3:4:0 |
| Liver Specific Outcomes | | | |
| LOS in days  (Mean + SD) |  |  | 17.08 + 16.23  (0-46) |
| GI Bleed (Y:N) |  |  | 3:9 |
| Ascites (Y:N) |  |  | 9:3 |
| Average Number of Hospitalizations  (Mean + SD) (Range) |  |  | 3.17 + 3.86  (1-10) |
| Hepatic Encephalopathy (Y:N) |  |  | 9:3 |
| Cirrhosis (Y:N) |  |  | 7:5 |
| Mean Survival From Enrollment in Days  (Mean + SD) (Range) |  |  | 547.67 + 442.89  (41-1148) |
| MELD Score  (Mean + SD) (Range) |  |  | 22.47 + 5.64  (12-31) |
| AFP (>3:<3:Not Done) |  |  | 6:0:6 |
| UTI (Y:N) |  |  | 0:12 |
| Sepsis (Y:N) |  |  | 1:11 |
| Pneumonia (Y:N) |  |  | 2:10 |
| SBP (Y:N) |  |  | 6:6 |

**a** p<0.05 AH vs. HC **b** p<0.001 AH vs. HC, HD; **c** p<0.001 AH vs. HC; **d** AH vs. HD p<0.01; **e** AH vs HC p<0.010

**Abbreviations:** **A:** Asian, **AA:** African American, **AFP:** alpha fetoprotein, **AH:** alcohol associated hepatitis, **dL:** deciliter, **g:** grams, **GI:** gastrointestinal; **HC:** healthy control, **HD:** healthy heavy drinker, **kg:** kilograms, **LOS:** Length of Stay, **m:** meters, **MELD:** model for end stage liver disease, **mg:** milligrams, **mmol:** millimole; **N:** No, **SBP:** spontaneous bacterial peritonitis, **SD:** Standard Deviation, **U:** Unknown, **U/L:** units per liter, **uL:** microliter, **UTI:**

urinary tract infection, **W:** White, **Y:** Yes

**S.Table 16. Patient details for the Validation Cohort (intact cell respiration)**

|  | **HC** | **HD** | **AH** |
| --- | --- | --- | --- |
| Number | 7 |  | 10 |
| Male:Female | 3:4 |  | 6:4 |
| Age in years (mean + SD) | 37.14 + 5.73 |  | 48.10 + 9.90^*^ |
| Alive:Dead | 7:0 |  | 3:7 |
| Race W:AA:A:U | 5:0:2:0 |  | 6:3:0:1 |
| Body mass index (kg/m^2^) | 22.24 + 3.91 |  | 31.09 + 7.27^**^ |
| Total leucocyte count (x1000/ul.) | 6.99 + 1.47 |  | 18.16 + 11.25^*^ |
| Platelet (x1000/ul.) | 277.57 + 99.47 |  | 132.20 + 76.73^**^ |
| Alanine amino transferase (U/L) | 15.88 + 7.86 |  | 58.00 + 27.05^***^ |
| Aspartate aminotransferase (U/L) | 18.14 + 2.91 |  | 140.60 + 68.54^***^ |
| Serum albumin (g/dL) | 4.40 + 0.25 |  | 2.98 + 0.40^***^ |
| Serum total protein (g/dL) | 7.07 + 0.41 |  | 5.50 + 0.93^***^ |
| Bilirubin (mg/dL) | 0.43 + 0.13 |  | 18.68 + 14.36^**^ |
| Alkaline phosphatase (U/L) | 63.29 + 32.04 |  | 163.50 + 97.24^*^ |
| Serum creatinine (mg/dL) | 0.77 + 0.15 |  | 1.97 + 1.98 |
| Blood urea nitrogen (mg/dL) | 11.14 + 3.34 |  | 38.00 + 33.21^*^ |
| Serum sodium (mmol/L) | 137.71 + 1.98 |  | 133.50 + 4.17^*^ |
| International normalized ratio | 0.95 + 0.07 |  | 2.22 + 0.87 |
| Steroids (Y:N) | 0:7 |  | 7:3 |
| Antibiotics (Y:N) | 1:6 |  | 10:0 |
| Antibiotics Count | 0.9 + 2.3 |  | 14.9 + 12.4 |
| Tobacco Use  Yes:Quit:Never:Unknown | 0:0:1:6 |  | 3:4:1:2 |
| Liver Specific Outcomes | | | |
| LOS in days  (Range + SD) |  |  | 16 + 14 |
| GI Bleed (Y:N) |  |  | 7:3 |
| Ascites (Y:N) |  |  | 6:4 |
| Average Number of Hospitalizations (mean + SD) (Range) |  |  | 2.30 + 2.98  (0-8) |
| Hepatic Encephalopathy (Y:N) |  |  | 8:2 |
| Cirrhosis (Y:N) |  |  | 8:2 |
| Mean Survival From Enrollment in Days  (Mean + SD) (Range) |  |  | 348.30 + 266.87  (5-711) |
| MELD Score  (Mean + SD) (Range) |  |  | 28.33 + 10.16  (10-45) |
| AFP (>3:<3:Not Done) |  |  | 1:4:5 |
| UTI (Y:N) |  |  | 0:10 |
| Sepsis (Y:N) |  |  | 2:8 |
| Pneumonia (Y:N) |  |  | 1:9 |
| SBP (Y:N) |  |  | 0:10 |

*p<0.05; ****** p<0.01; *** p<0.001.

**Abbreviations:** **A:** Asian, **AA:** African American, **AFP:** alpha fetoprotein, **AH:** alcohol associated hepatitis, **dL:** deciliter, **g:** grams, **GI:** gastrointestinal; **HC:** healthy control, **HD:** healthy heavy drinker, **kg:** kilograms, **LOS:** Length of Stay, **m:** meters, **MELD:** model for end stage liver disease, **mg:** milligrams, **mmol:** millimole; **N:** No, **SBP:** spontaneous bacterial peritonitis, **SD:** Standard Deviation, **U:** Unknown, **U/L:** units per liter, **uL:** microliter, **UTI:**

urinary tract infection, **W:** White, **Y:** Yes

**S.Table 17. Patient details for the Validation Cohort (permeabilized cells**)

|  | HC | HD | AH |
| --- | --- | --- | --- |
| Number | 7 |  | 10 |
| Male:Female | 3:4 |  | 6:4 |
| Age in years (mean + SD) | 37.14 + 5.73 |  | 48.10 + 9.90^*^ |
| Alive:Dead | 7:0 |  | 3:7 |
| Race W:AA:A:U | 5:0:2:0 |  | 6:3:0:1 |
| Body mass index (kg/m^2^) | 22.24 + 3.91 |  | 31.09 + 7.27^**^ |
| Total leucocyte count (x1000/ul.) | 6.99 + 1.47 |  | 18.16 + 11.25^*^ |
| Platelet (x1000/ul.) | 277.57 + 99.47 |  | 132.20 + 76.73^**^ |
| Alanine amino transferase (U/L) | 15.88 + 7.86 |  | 58.00 + 27.05^***^ |
| Aspartate aminotransferase (U/L) | 18.14 + 2.91 |  | 140.60 + 68.54^***^ |
| Serum albumin (g/dL) | 4.40 + 0.25 |  | 2.98 + 0.40^***^ |
| Serum total protein (g/dL) | 7.07 + 0.41 |  | 5.50 + 0.93^***^ |
| Bilirubin (mg/dL) | 0.43 + 0.13 |  | 18.68 + 14.36^**^ |
| Alkaline phosphatase (U/L) | 63.29 + 32.04 |  | 163.50 + 97.24^*^ |
| Serum creatinine (mg/dL) | 0.77 + 0.15 |  | 1.97 + 1.98 |
| Blood urea nitrogen (mg/dL) | 11.14 + 3.34 |  | 38.00 + 33.21^*^ |
| Serum sodium (mmol/L) | 137.71 + 1.98 |  | 133.50 + 4.17^*^ |
| International normalized ratio | 0.95 + 0.07 |  | 2.22 + 0.87 |
| Steroids (Y:N) | 0:7 |  | 7:3 |
| Antibiotics (Y:N) | 1:6 |  | 10:0 |
| Antibiotics Count | 0.9 + 2.3 |  | 14.9 + 12.4 |
| Tobacco Use  Yes:Quit:Never:Unknown | 0:0:1:6 |  | 3:4:1:2 |
| Liver Specific Outcomes | | | |
| LOS in days  (Range + SD) |  |  | 16 + 14 |
| GI Bleed (Y:N) |  |  | 7:3 |
| Ascites (Y:N) |  |  | 6:4 |
| Average Number of Hospitalizations (mean + SD) (Range) |  |  | 2.30 + 2.98  (0-8) |
| Hepatic Encephalopathy (Y:N) |  |  | 8:2 |
| Cirrhosis (Y:N) |  |  | 8:2 |
| Mean Survival From Enrollment in Days  (Mean + SD) (Range) |  |  | 348.30 + 266.87  (5-711) |
| MELD Score  (Mean + SD) (Range) |  |  | 28.33 + 10.16  (10-45) |
| AFP (>3:<3:Not Done) |  |  | 1:4:5 |
| UTI (Y:N) |  |  | 0:10 |
| Sepsis (Y:N) |  |  | 2:8 |
| Pneumonia (Y:N) |  |  | 1:9 |
| SBP (Y:N) |  |  | 0:10 |

*p<0.05; ****** p<0.01; *** p<0.001.

**Abbreviations:** **A:** Asian, **AA:** African American, **AFP:** alpha fetoprotein, **AH:** alcohol associated hepatitis, **dL:** deciliter, **g:** grams, **GI:** gastrointestinal; **HC:** healthy control, **HD:** healthy heavy drinker, **kg:** kilograms, **LOS:** Length of Stay, **m:** meters, **MELD:** model for end stage liver disease, **mg:** milligrams, **mmol:** millimole; **N:** No, **SBP:** spontaneous bacterial peritonitis, **SD:** Standard Deviation, **U:** Unknown, **U/L:** units per liter, **uL:** microliter, **UTI:** urinary tract infection, **W:** White, **Y:** Yes

**S.Table 18. Patient details for telomere length data**

|  | **HC** | **HD** | **AH** |
| --- | --- | --- | --- |
| Number | 13 | 8 | 72 |
| Male:Female | 10:3 | 5:3 | 46:26 |
| Age in years (mean + SD) | 45.69 + 12.77 | 40.75 + 15.30 | 47.94 + 11.44 |
| Alive:Dead | 13:0 | 8:0 | 35:37 |
| Race W:AA:A:U | 6:1:3:3 | 6:1:0:1 | 56:11:1:4 |
| Body mass index (kg/m^2^) | 25.56 + 4.80 | 28.95 + 5.67 | 28.79 + 3.10 |
| Total leucocyte count (x1000/ul.) | 6.41 + 1.97 | 6.54 + 2.81 | 13.87 + 7.69^a,b^ |
| Platelet (x1000/ul.) | 256.09 + 86.82 | 262.71 + 67.99 | 121.78 + 73.35 ^c^ |
| Alanine amino transferase (U/L) | 19.27 + 8.93 | 34.57 + 18.61 | 52.39 + 42.98 ^d^ |
| Aspartate aminotransferase (U/L) | 20.63 + 6.07 | 25.00 + 4.58 | 131.51 + 98.55^e,f^ |
| Serum albumin (g/dL) | 4.37 + 0.26 | 4.51 + 0.34 | 2.96 + 0.55^c^ |
| Serum total protein (g/dL) | 7.19 + 0.37 | 7.36 + 0.36 | 5.67 + 0.89^c^ |
| Bilirubin (mg/dL) | 0.79 + 0.89 | 0.36 + 0.14 | 15.70 + 11.98^e,f^ |
| Alkaline phosphatase (U/L) | 69.91 + 20.93 | 63.57 + 23.54 | 175.08 + 131.57^d,^ |
| Serum creatinine (mg/dL) | 0.81 + 0.14 | 0.84 + 0.08 | 1.87 + 1.71 |
| Blood urea nitrogen (mg/dL) | 12.91 + 4.21 | 12.29 + 3.35 | 29.67 + 26.56 |
| Serum sodium (mmol/L) | 139.45 + 1.69 | 138.86 + 2.67 | 133.85 + 5.08 ^e,b^ |
| International normalized ratio | 1.02 + 0.12 | 1.09 + 0.23 | 1.87 + 0.52^c^ |
| Steroids (Y:N) | 0:13 | 0:8 | 42:30 |
| Antibiotics (Y:N) | 4:9 | 1:7 | 50:12 |
| Antibiotics Count | 2.3 + 6.6 | 2.1 + 6.0 | 16.2 + 15.9 |
| Tobacco Use  Yes:Quit:Never:Unknown | 0:0:6:7 | 1:0:2:5 | 18:28:22:4 |
| Liver Specific Outcomes | | | |
| LOS in days  (Mean + SD) |  |  | 19 + 19 |
| GI Bleed (Y:N) |  |  | 27:45 |
| Ascites (Y:N) |  |  | 50:22 |
| Average Number of Hospitalizations (Mean + SD) (Range) |  |  | 1.76 + 2.38  (0-10) |
| Hepatic Encephalopathy (Y:N) |  |  | 52:20 |
| Cirrhosis (Y:N) |  |  | 51:21 |
| Mean Survival From Enrollment in Days  (Mean + SD) (Range) |  |  | 402.92 + 317.56  (5-1341) |
| MELD Score  (Mean + SD) (Range) |  |  | 26.42 + 8.86  (8.33-52.82) |
| AFP (>3:<3:Not Done) |  |  | 18:21:33 |
| UTI (Y:N) |  |  | 0:72 |
| Sepsis (Y:N) |  |  | 7:65 |
| Pneumonia (Y:N) |  |  | 4:68 |
| SBP (Y:N) |  |  | 14:58 |

**a** p<0.01 AH vs. HC; **b** p<0.05 AH vs. HD; **c** p<0.001 AH vs. HC, HD; **d** AH vs. HC p<0.05; **e** AH vs HC p<0.001; **f** AH vs. HD p<0.01

**Abbreviations:** **A:** Asian, **AA:** African American, **AFP:** alpha fetoprotein, **AH:** alcohol associated hepatitis, **dL:** deciliter, **g:** grams, **GI:** gastrointestinal; **HC:** healthy control, **HD:** healthy heavy drinker, **kg:** kilograms, **LOS:** Length of Stay, **m:** meters, **MELD:** model for end stage liver disease, **mg:** milligrams, **mmol:** millimole; **N:** No, **SBP:** spontaneous bacterial peritonitis, **SD:** Standard Deviation, **U:** Unknown, **U/L:** units per liter, **uL:** microliter, **UTI:** urinary tract infection, **W:** White, **Y:** Yes

**S.Table 19. Patient details for plasma tricarboxylic acid cycle intermediate concentrations**

|  | **HC** | **HD** | **AH** |
| --- | --- | --- | --- |
| Number | 27 | 8 | 59 |
| Male:Female | 15:12 | 5:3 | 38:21 |
| Age in years (mean + SD) | 42.00 + 13.82 | 46.97 + 11.93 | 46.97 + 11.93^a^ |
| Alive:Dead | 27:0 | 8:0 | 32:27 |
| Race W:AA:A:U | 18:1:5:3 | 7:1:0:0 | 47:7:1:4 |
| Body mass index (kg/m^2^) | 24.80 + 4.76 | 27.09 + 2.83 | 27.40 + 5.61^a^ |
| Total leucocyte count (x1000/ul.) | 6.90 + 3.26 | 5.56 + 1.31 | 14.01 + 7.83^b,c^ |
| Platelet (x1000/ul.) | 264.13 + 74.33 | 235.00 + 45.74 | 128.64 + 70.84^d^ |
| Alanine amino transferase (U/L) | 18.22 + 8.17 | 28.63 + 19.57 | 49.86 + 40.24^b^ |
| Aspartate aminotransferase (U/L) | 19.78 + 4.85 | 27.13+ 16.31 | 131.49 + 97.42^b,c^ |
| Serum albumin (g/dL) | 4.47 + 0.33 | 4.48 + 0.33 | 3.05 + 0.57 ^d^ |
| Serum total protein (g/dL) | 7.22 + 0.38 | 7.23 + 0.41 | 5.78 + 0.95^d^ |
| Bilirubin (mg/dL) | 0.63 + 0.66 | 0.31 + 0.08 | 16.78 + 12.40^d^ |
| Alkaline phosphatase (U/L) | 66.26 + 24.00 | 75.25 + 42.62 | 182.07 + 142.58^b,e^ |
| Serum creatinine (mg/dL) | 0.84 + 0.17 | 0.79 + 0.12 | 1.87 + 1.67^c^ |
| Blood urea nitrogen (mg/dL) | 12.91 + 3.49 | 10.75 + 3.01 | 29.24 + 26.99^c^ |
| Serum sodium (mmol/L) | 139.39 + 2.33 | 138.00 + 2.27 | 134.34 + 4.90^b^ |
| International normalized ratio | 1.01 + 0.09 | 1.06 + 0.22 | 1.84 + 0.62^d^ |
| Steroids (Y:N) | 1:26 | 0:8 | 33:26 |
| Antibiotics (Y:N) | 4:23 | 2:6 | 43:16 |
| Antibiotics Count | 1.6 + 5.6 | 2.2 + 6.0 | 16.6 + 16.4 |
| Tobacco Use  Yes:Quit:Never:Unknown | 0:1:9:17 | 1:1:1:5 | 14:22:19:4 |
| Liver Specific Outcomes | | | |
| LOS in days  (Mean + SD) |  |  | 18.43 + 19.36  (0-92) |
| GI Bleed (Y:N) |  |  | 22:37 |
| Ascites (Y:N) |  |  | 44:15 |
| Average Number of Hospitalizations  (Mean + SD) (Range) |  |  | 2.15 + 2.72  (0-10) |
| Hepatic Encephalopathy (Y:N) |  |  | 43:16 |
| Cirrhosis (Y:N) |  |  | 41:18 |
| Mean Survival From Enrollment in Days  (Mean + SD) (Range) |  |  | 432.63 + 400.24  (5-1755) |
| MELD Score  (Mean + SD) (Range) |  |  | 26.47 + 9.04  (8.33-52.82) |
| AFP (>3:<3:Not Done) |  |  | 14:15:30 |
| UTI (Y:N) |  |  | 1:58 |
| Sepsis (Y:N) |  |  | 6:53 |
| Pneumonia (Y:N) |  |  | 3:56 |
| SBP (Y:N) |  |  | 13:46 |

**a** p<0.05 AH vs. HC; **b** p<0.001 AH vs. HC; **c** p<0.01 AH vs. HD; **d** AH vs. HC, HD p<0.001; **e** AH vs HD <0.05;

**Abbreviations:** **A:** Asian, **AA:** African American, **AFP:** alpha fetoprotein, **AH:** alcohol associated hepatitis, **dL:** deciliter, **g:** grams, **GI:** gastrointestinal; **HC:** healthy control, **HD:** healthy heavy drinker, **kg:** kilograms, **LOS:** Length of Stay, **m:** meters, **MELD:** model for end stage liver disease, **mg:** milligrams, **mmol:** millimole; **N:** No, **SBP:** spontaneous bacterial peritonitis, **SD:** Standard Deviation, **U:** Unknown, **TCA:** tricarboxylic acid cycle, **U/L:** units per liter, **uL:** microliter, **UTI:** urinary tract infection, **W:** White, **Y:** Yes

**S.Table 20. Patient details for tricarboxylic acid cycle intermediate concentrations in PBMC**

|  | **HC** | **HD** | **AH** |
| --- | --- | --- | --- |
| Number | 14 | 8 | 69 |
| Male:Female | 8:6 | 5:3 | 45:24 |
| Age in years (mean + SD) | 37.50 + 13.07 | 46.00 + 13.82 | 46.93 + 11.77^a^ |
| Alive:Dead | 14:0 | 8:0 | 34:35 |
| Race W:AA:A:U | 7:1:4:2 | 7:1:0:0 | 54:10:1:4 |
| Body mass index (kg/m^2^) | 23.95 + 5.25 | 27.09 + 2.83 | 29.04 + 6.26^a^ |
| Total leucocyte count (x1000/ul.) | 7.94 + 4.02 | 5.56 + 1.31 | 14.04 + 7.56^a,b^ |
| Platelet (x1000/ul.) | 271.00 + 90.39 | 235.00 + 45.74 | 125.09 + 70.59^c^ |
| Alanine amino transferase (U/L) | 20.58 + 10.40 | 28.63 + 19.57 | 55.81 + 47.89^a^ |
| Aspartate aminotransferase (U/L) | 19.67 + 3.11 | 27.13 + 16.31 | 134.88 + 102.47^d,b^ |
| Serum albumin (g/dL) | 4.51 + 0.36 | 4.48 + 0.33 | 3.01 + 0.56^c^ |
| Serum total protein (g/dL) | 7.24 + 0.35 | 7.23 + 0.41 | 5.71 + 0.91^c^ |
| Bilirubin (mg/dL) | 0.48 + 0.17 | 0.31 + 0.08 | 16.55 + 12.50^c^ |
| Alkaline phosphatase (U/L) | 62.00 + 25.19 | 75.25 + 42.62 | 173.91 + 134.00^e^ |
| Serum creatinine (mg/dL) | 0.81 + 0.18 | 0.79 + 0.12 | 1.86 + 1.60 |
| Blood urea nitrogen (mg/dL) | 12.25 + 3.67 | 10.75 + 3.01 | 30.22 + 26.30^a^ |
| Serum sodium (mmol/L) | 138.83 + 2.20 | 138.00 + 2.27 | 134.41 + 5.06^e^ |
| International normalized ratio | 1.04 + 0.15 | 1.06 + 0.22 | 1.86 + 0.58^e,f^ |
| Steroids (Y:N) | 1:13 | 0:8 | 40:29 |
| Antibiotics (Y:N) | 2:12 | 2:6 | 11:58 |
| Antibiotics Count | 2.4 + 6.5 | 2.3 + 6.0 | 17.9 + 17.0 |
| Tobacco Use  Yes:Quit:Never:Unknown | 0:0:2:12 | 1:1:1:5 | 16:26:22:5 |
| Liver Specific Outcomes | | | |
| LOS in days  (Mean + SD) |  |  | 18.54 + 18.39  (0-92) |
| GI Bleed (Y:N) |  |  | 26:43 |
| Ascites (Y:N) |  |  | 49:20 |
| Average Number of Hospitalizations (Mean + SD) (Range) |  |  | 1.94 + 2.59  (0-10) |
| Hepatic Encephalopathy (Y:N) |  |  | 50:19 |
| Cirrhosis (Y:N) |  |  | 48:21 |
| Mean Survival From Enrollment in Days  (mean + SD) (Range) |  |  | 417.78 + 395.80  (5-1755) |
| MELD Score  (Mean + SD) (Range) |  |  | 26.54 + 9.07  (8.83-52.82) |
| AFP (>3:<3:Not Done) |  |  | 19:17:33 |
| UTI (Y:N) |  |  | 1:68 |
| Sepsis (Y:N) |  |  | 7:62 |
| Pneumonia (Y:N) |  |  | 4:65 |
| SBP (Y:N) |  |  | 15:54 |

**a** p<0.05 AH vs. HC; **b** p<0.01 AH vs. HD; **c** AH vs. HC, HD p<0.001; **d** AH vs HC <0.001; **e** AH vs HC <0.01, **f** AH vs HD <0.001

**Abbreviations:** **A:** Asian, **AA:** African American, **AFP:** alpha fetoprotein, **AH:** alcohol associated hepatitis, **dL:** deciliter, **g:** grams, **GI:** gastrointestinal; **HC:** healthy control, **HD:** healthy heavy drinker, **kg:** kilograms, **LOS:** Length of Stay, **m:** meters, **MELD:** model for end stage liver disease, **mg:** milligrams, **mmol:** millimole; **N:** No, **PBMC:** peripheral blood mononuclear cells, **SBP:** spontaneous bacterial peritonitis, **SD:** Standard Deviation, **U:** Unknown, **TCA:** tricarboxylic acid cycle, **U/L:** units per liter, **uL:** microliter, **UTI:** urinary tract infection, **W:** White, **Y:** Yes

**S.Table 21. Patient details for tricarboxylic acid cycle intermediate concentrations in PBMC (subanalysis of AH only)**

|  | **AH** |
| --- | --- |
| Number | 69 |
| Male:Female | 45:24 |
| Age in years (mean + SD) | 46.93 + 11.77 |
| Alive:Dead | 34:35 |
| Race W:AA:A:U | 54:10:1:4 |
| Body mass index (kg/m^2^) | 29.04 + 6.26 |
| Total leucocyte count (x1000/ul.) | 14.04 + 7.56 |
| Platelet (x1000/ul.) | 125.09 + 70.59 |
| Alanine amino transferase (U/L) | 55.81 + 47.89 |
| Aspartate aminotransferase (U/L) | 134.88 + 102.47 |
| Serum albumin (g/dL) | 3.01 + 0.56 |
| Serum total protein (g/dL) | 5.71 + 0.91 |
| Bilirubin (mg/dL) | 16.55 + 12.50 |
| Alkaline phosphatase (U/L) | 173.91 + 134.00 |
| Serum creatinine (mg/dL) | 1.86 + 1.60 |
| Blood urea nitrogen (mg/dL) | 30.22 + 26.30 |
| Serum sodium (mmol/L) | 134.41 + 5.06 |
| International normalized ratio | 1.86 + 0.58 |
| Steroids (Y:N) | 40:29 |
| Antibiotics (Y:N) | 11:58 |
| Antibiotics Count | 17.9 + 17.0 |
| Tobacco Use  Yes:Quit:Never:Unknown |  |
| LOS in days  (Mean + SD) | 18.54 + 18.39  (0-92) |
| GI Bleed (Y:N) | 26:43 |
| Ascites (Y:N) | 49:20 |
| Average Number of Hospitalizations  (Mean + SD) (Range) | 1.94 + 2.59  (0-10) |
| Hepatic Encephalopathy (Y:N) | 50:19 |
| Cirrhosis (Y:N) | 48:21 |
| Mean Survival From Enrollment in Days  (Mean + SD) (Range) | 417.78 + 395.80  (5-1755) |
| MELD Score  (Mean + SD) (Range) | 26.54 + 9.07  (8.83-52.82) |
| AFP (>3:<3:Not Done) | 19:17:33 |
| UTI (Y:N) | 1:68 |
| Sepsis (Y:N) | 7:62 |
| Pneumonia (Y:N) | 4:65 |
| SBP (Y:N) | 15:54 |

**Abbreviations:** **A:** Asian, **AA:** African American, **AFP:** alpha fetoprotein, **AH:** alcohol associated hepatitis, **dL:** deciliter, **g:** grams, **GI:** gastrointestinal; **HC:** healthy control, **HD:** healthy heavy drinker, **kg:** kilograms, **LOS:** Length of Stay, **m:** meters, **MELD:** model for end stage liver disease, **mg:** milligrams, **mmol:** millimole; **N:** No, **PBMC:** peripheral blood mononuclear cells, **SBP:** spontaneous bacterial peritonitis, **SD:** Standard Deviation, **U:** Unknown, **TCA:** tricarboxylic acid cycle, **U/L:** units per liter, **uL:** microliter, **UTI:** urinary tract infection, **W:** White, **Y:** Yes

**S.Table 22. Patient details for fatigue data (from PROMIS questionnaire)**

|  | **HC** | **HD** | **AH** |
| --- | --- | --- | --- |
| Number | 17 | 3 | 12 |
| Male:Female | 10:7 | 2:1 | 7:5 |
| Age in years (mean + SD) | 42.59 + 14.77 | 42.67 + 11.06 | 50.75 + 13.18 |
| Alive:Dead | 17:0 | 3:0 | 3:9 |
| Race W:AA:A:U | 12:0:3:2 | 3:0:0:0 | 9:2:0:1 |
| Body mass index (kg/m^2^) | 26.22 + 4.87 | 28.54 + 3.03 | 29.95 + 5.14 |
| Total leucocyte count (x1000/ul.) | 6.50 + 1.67 | 5.80 + 1.57 | 14.07 + 9.56^a^ |
| Platelet (x1000/ul.) | 250.15 + 68.28 | 222.33 + 60.17 | 102.42 + 75.39^b,c^ |
| Alanine amino transferase (U/L) | 18.77 + 6.00 | 34.67 + 34.30 | 47.42 + 28.29^d^ |
| Aspartate aminotransferase (U/L) | 20.92 + 5.16 | 21.33 + 7.51 | 110.17 + 44.84^e^ |
| Serum albumin (g/dL) | 4.46+ 0.28 | 4.37 + 0.15 | 2.89 + 0.59^e^ |
| Serum total protein (g/dL) | 7.20 + 0.41 | 7.07 + 0.60 | 5.59 + 0.90^b,f^ |
| Bilirubin (mg/dL) | 0.71 + 0.86 | 0.33 + 0.06 | 14.67 + 12.15^b,c^ |
| Alkaline phosphatase (U/L) | 69.15 + 23.81 | 77.00 + 26.85 | 124.92 + 58.77^d^ |
| Serum creatinine (mg/dL) | 0.88 + 0.19 | 0.80 + 0.04 | 2.00 + 2.14 |
| Blood urea nitrogen (mg/dL) | 12.46 + 2.73 | 11.33 + 2.89 | 34.50 + 31.92^a^ |
| Serum sodium (mmol/L) | 139.46 + 2.50 | 137.33 + 2.08 | 133.17 + 5.27^b^ |
| International normalized ratio | 0.98 + 0.04 | 1.00 + 0.0 | 2.13 + 0.65^b,f^ |
| Steroids (Y:N) | 0:17 | 0:3 | 7:5 |
| Antibiotics (Y:N) | 3:14 | 1:2 | 12:0 |
| Antibiotics Count | 1.2 + 3.7 | 0.3 + 0.6 | 19.0 + 18.9 |
| Tobacco Use  Yes:Quit:Never:Unknown | 0:1:6:10 | 0:1:0:2 | 2:6:2:2 |
| Liver Specific Outcomes | | | |
| LOS in days  (Mean + SD) |  |  | 19.75 + 14.46  (0-46) |
| GI Bleed (Y:N) |  |  | 6:6 |
| Ascites (Y:N) |  |  | 9:3 |
| Average Number of Hospitalizations  (Mean + SD) (Range) |  |  | 3.0 + 3.43  (0-10) |
| Hepatic Encephalopathy (Y:N) |  |  | 10:2 |
| Cirrhosis (Y:N) |  |  | 10:2 |
| Mean Survival From Enrollment in Days  (Mean + SD) (Range) |  |  | 384.00 + 301.31  (5-1026) |
| MELD Score  (Mean + SD) (Range) |  |  | 27.63 + 8.10  (15-45) |
| UTI (Y:N) |  |  | 0:12 |
| Sepsis (Y:N) |  |  | 2:10 |
| Pneumonia (Y:N) |  |  | 1:11 |
| SBP (Y:N) |  |  | 3:9 |

**a** p<0.05 AH vs. HC; **b** p<0.001 AH vs. HC; **c** AH vs. HD p<0.05; **d** AH vs HC <0.01; **e** AH vs HC, HD <0.001, **f** AH vs HD <0.01

**Abbreviations:** **A:** Asian, **AA:** African American, **AFP:** alpha fetoprotein, **AH:** alcohol associated hepatitis, **dL:** deciliter, **g:** grams, **GI:** gastrointestinal; **HC:** healthy control, **HD:** healthy heavy drinker, **kg:** kilograms, **LOS:** Length of Stay, **m:** meters, **MELD:** model for end stage liver disease, **mg:** milligrams, **mmol:** millimole; **N:** No, **SBP:** spontaneous bacterial peritonitis, **SD:** Standard Deviation, **U:** Unknown, **U/L:** units per liter, **uL:** microliter, **UTI:** urinary tract infection, **W:** White, **Y:** Yes

**S.Table 23. Survivors vs Non-Survivors in AH**

|  | **Survivors** | **Non-Survivors** |
| --- | --- | --- |
| Number | 40 | 41 |
| Male:Female | 24:16 | 28:13 |
| Age in years (mean + SD) | 48.50 + 12.16 | 47.37 + 11.04 |
| Race W:AA:A:U | 35:3:1:1 | 29:9:0:3 |
| Body mass index (kg/m^2^) | 26.31 + 4.64 | 30.88 +6.65^***^ |
| Total leucocyte count (x1000/ul.) | 12.55 + 7.69 | 14.81 + 7.49 |
| Platelet (x1000/ul.) | 144.05 + 71.60 | 107.29 + 67.80^*^ |
| Alanine amino transferase (U/L) | 52.15 + 48.53 | 55.02 + 41.67 |
| Aspartate aminotransferase (U/L) | 119.20 *+* 103.58 | 140.68 + 91.38 |
| Serum albumin (g/dL) | 3.08 + 0.48 | 2.87 + 0.58 |
| Serum total protein (g/dL) | 5.91 + 1.00 | 5.46 + 0.46^*^ |
| Bilirubin (mg/dL) | 14.87 + 12.40 | 16.43 + 11.73 |
| Alkaline phosphatase (U/L) | 199.73 + 166.54 | 148.73 + 67.45 |
| Serum creatinine (mg/dL) | 1.48 + 1.08 | 2.21 + 2.01^*^ |
| Blood urea nitrogen (mg/dL) | 26.25 + 22.79 | 32.90 + 28.71 |
| Serum sodium (mmol/L) | 134.88 + 4.72 | 133.37 + 5.22 |
| International normalized ratio | 1.75 + 0.65 | 1.99 + 0.54 |
| Steroids (Y:N) | 23:17 | 24:17 |
| Tobacco Use  Yes:Quit:Never:Unknown | 9:10:20:1 | 10:21:5:5 |
| Antibiotics (Y:N) | 31:9 | 37:4 |
| LOS in days  (Mean + SD) | 15.93 + 17.99  (0-92) | 19.85 + 16.98  (2-88) |
| GI Bleed (Y:N) | 16:24 | 17:24 |
| Ascites (Y:N) | 28:12 | 28:13 |
| Hepatic Encephalopathy (Y:N) | 27:13 | 32:9 |
| Cirrhosis (Y:N) | 25:15 | 31:10 |
| Mean Survival From Enrollment in Days  (Mean + SD) (Range) | 622.82 + 424.29  (155-1755) | 247.78 + 198.68^***^  (1-836) |
| MELD Score  (Mean + SD) (Range) | 23.71 + 8.37  (8.33-39.66) | 28.79 + 7.80^**^  (10.30-52.82) |
| AFP (>3:<3:Not Done) | 9:10:21 | 14:13:14 |
| UTI (Y:N) | 1:39 | 0:41 |
| Sepsis (Y:N) | 2:38 | 6:35 |
| Pneumonia (Y:N) | 1:39 | 4:37 |
| SBP (Y:N) | 8:32 | 8:33 |

*p<0.05; ****** p<0.01; *** p<0.001.

**Abbreviations:** **A:** Asian, **AA:** African American, **AFP:** alpha fetoprotein, **AH:** alcohol associated hepatitis, **dL:** deciliter, **g:** grams, **GI:** gastrointestinal; **HC:** healthy control, **HD:** healthy heavy drinker, **kg:** kilograms, **LOS:** Length of Stay, **m:** meters, **MELD:** model for end stage liver disease, **mg:** milligrams, **mmol:** millimole; **N:** No, **SBP:** spontaneous bacterial peritonitis, **SD:** Standard Deviation, **U:** Unknown, **U/L:** units per liter, **uL:** microliter, **UTI:** urinary tract infection, **W:** White, **Y:** Yes

**S.Table 24**. Clinical and laboratory findings in patients with elevated intermediary metabolites in peripheral blood mononuclear cells from patients with alcohol associated hepatitis

|  | **Not high TCA** | **High TCA** | **p** |
| --- | --- | --- | --- |
| Number of subjects | 58 | 12 |  |
| Age (years) | 47.11 (11.47) | 44.91 (12.90) | 0.571 |
| Death (%) | 27 (47.4) | 6 (54.5) | 0.915 |
| Body mass index | 28.90 (6.99) | 28.21 (5.49) | 0.759 |
| Length of stay in hospital (days) | 19.72 (19.89) | 16.44 (15.63) | 0.642 |
| GI_Bleed (%) | 19 (32.8) | 5 (41.7) | 0.797 |
| Ascites (%) | 36 (62.1) | 9 (75) | 1 |
| Spontaneous bacterial peritonitis (%) | 8 (13.8) | 6 (50.0) | 0.014 |
| Number of hospitalizations | 1.63 (2.48) | 3.36 (3.14) | 0.046 |
| Hepatic encephalopathy (%) | 28 (48.3) | 7 (58.3) | 0.701 |
| Cirrhosis (%) | 42 (72.4) | 5 (41.7) | 0.644 |
| Total leucocyte count (x1000/μL) | 13.21 (7.16) | 13.68 (7.00) | 0.841 |
| Platelet (x1000/μL) | 122.36 (71.31) | 136.55 (78.99) | 0.555 |
| Serum alanine amino transferase (U/L) | 58.84 (51.29) | 31.45 (14.21) | 0.085 |
| Serum aspartate amino transferase (U/L) | 143.68 (108.31) | 105.36 (71.96) | 0.266 |
| Serum albumin (g/dL) | 2.99 (0.55) | 2.99 (0.68) | 0.984 |
| Serum total protein (g/dL) | 5.73 (0.87) | 6.15 (1.10) | 0.17 |
| Serum bilirubin (mg/dL) | 14.72 (12.74) | 11.91 (7.74) | 0.484 |
| Serum alkaline phosphatase (U/L) | 180.98 (148.10) | 164.45 (93.04) | 0.723 |
| Serum creatinine (mg/dL) | 1.79 (1.44) | 1.51 (1.84) | 0.58 |
| Blood urea nitrogen (mg/dL) | 29.80 (25.33) | 24.45 (26.19) | 0.526 |
| International normalized ratio | 1.87 (0.50) | 2.10 (0.97) | 0.262 |
| MELD (mean(SD)) | 26.48 (9.54) | 26.81 (6.71) | 0.911 |
| Telomere length (kB) | 8.83 (4.62) | 13.80 (2.53) | 0.007 |
| Log telomere length | -0.25 (0.04) | -0.30 (0.06) | 0.005 |

Numbers in parentheses are mean±standard deviation (unless specified)

| **Gene Set** | **Role** |
| --- | --- |
| Telomere Length | Genes related to the length of the telomeres^35, 36^ |
| Shelterin Complex | Protect telomeres from DNA repair, if shelterin is not present the DNA Repair mechanisms will recognize the telomeres as damaged DNA^36, 37^ |
| Shelterin Binding Genes | Regulate the binding of the shelterin protein complex to the telomeres^36-38^ |
| Telomerase Localization | Regulate the binding of telomerase (enzyme that extends the 3’ end of telomere DNA) to the telomere^39^ |
| DNA Repair | Genes involved in the DNA Repair Pathway^40^ |
| ALT Enhancing | Alternate Lengthening of Telomeres (ALT) lengthens telomeres when telomerase is not present in the cell- ALT enhancing genes enhance activity of ALT^40, 41^ |
| ALT Repressing | Opposes ALT enhancing gene effects^41^ |
| Sumolyation | Regulator of telomere length – enhances shelterin binding and prevents telomerase binding^42^ |
| TERRA | RNA that is transcribed from telomeres functions with the shelterin complex to inhibit binding of telomerase.^36, 37^ |
| Extrachromosomal Repeat Binding | These are repeat DNA sequences that accumulate on telomeres that are not protected by DNA and interfere with binding ability of other molecules (shelterin and telomerase)^40, 41^ |
| Telomere Localization | Genes that are related to localizing the telomere to shelterin, telomerase and others that bind to telomeres^37^ |

**S.Table 25. Genes involved in telomere length maintenance**.
